# Tas2r105 ameliorates gut inflammation, possibly through influencing the gut microbiota and metabolites

**DOI:** 10.1128/msystems.01556-24

**Published:** 2025-03-13

**Authors:** Xiucai Lan, Liang Ma, Jiaming Ma, Zhipeng Huang, Lingling Liu, Feng Li, Mingbang Wang, Yaomin Hu

**Affiliations:** 1 Department of Geriatrics, Renji Hospital, School of Medicine, Shanghai Jiaotong University 12474 , Shanghai, China; 2 Department of Radiology, Children's Hospital of Fudan University, National Children's Medical Center 643902 , Shanghai, China; 3 Department of Health-Related Product Assessment, Shanghai Municipal Center for Disease Control and Prevention 117753 , Shanghai, China; 4 Departments of Gastroenterology, First Hospital of Quanzhou affiliated to Fujian Medical University, Quanzhou, China; 5 Department of Laboratory Animal Science, Shanghai Public Health Clinical Center 34748 , Shanghai, China; 6 Department of Neonatology, Affiliated Shenzhen Women and Children's Hospital (Longgang) of Shantou University Medical College (Longgang District Maternity & Child Healthcare Hospital of Shenzhen City, Shenzhen, Guangdong, China; 7 Department of Experiment & Research, South China Hospital, Medical School, Shenzhen University 47890, Shenzhen, Guangdong, China; University of California San Diego, La Jolla, California, USA

**Keywords:** inflammatory bowel disease, Tas2r105, gut microbiota

## Abstract

Inflammatory bowel disease (IBD) is an immune-mediated gastrointestinal disorder that significantly impacts the life quality of people worldwide. Genetic factors play crucial roles in the development of IBD. Tas2rs, members of the G protein-coupled receptor (GPCR) superfamily, are known for their roles in bitter taste perception. However, Tas2rs have also been identified in the gut, where they help sense luminal contents and regulate gastrointestinal hormones. Periodontal Tas2r105 has been shown to modulate innate immunity by interacting with metabolites produced by oral bacteria. In this study, we observed increased Tas2r105 in the inflammatory colons induced by dextran sulfate sodium salt (DSS). We also noted that α-gustducin, the α-subunit of GPCRs, is present in the intestine, and that α-gustducin knockout mice exhibit aggravated colitis. Based on these findings, we hypothesize that Tas2r105 may play a role in immune regulation during IBD pathogenesis. To test this hypothesis, we used Tas2r105 knockout (KO) mice in a colitis model. Our results show that the KO mice had significantly shorter colon length, more severe colon inflammation, and greater destruction of the gut barrier compared with control mice. We also observed increased recruitment of macrophages to the lamina propria mucosa in the KO mice. Microbiological analysis revealed a significant increase in *Proteobacteria* and *Bacteroidota*, with a concomitant decrease in *Firmicutes* after Tas2r105 knockout. Metabolomic analysis showed a significant reduction in lysophosphatidylethanolamine (LPE) levels in the KO mice, which is known to have anti-inflammatory effects. Based on these findings, we speculate that Tas2r105 may help protect the intestine from inflammation by influencing the gut microbiota composition and LPE production.

**IMPORTANCE:**

Increased Tas2r105 was detected in the inflamed colon of mice outside the tongue. Tas2r105 deletion aggravated mice colon colitis. Tas2r105 might alleviate mice colitis by downregulating the *Proteobacteria* and the *Bacteroidota* abundance in the colon. Lysophosphatidylethanolamine (LPE) might be the key metabolite that mediated the intestinal protection of Tas2r105.

## INTRODUCTION

Inflammatory bowel disease (IBD) is an immune-related gastrointestinal disorder, which includes Crohn’s disease (CD) and ulcerative colitis (UC), and is characterized by chronic and progressive intestinal inflammation. In the late 20th century, IBD was considered a Western disease. However, in the 21st century, the incidence and prevalence of IBD have increased globally. Although still lower than in Western countries, the number of IBD cases in Asia is rising (1). Genetic susceptibility and environmental factors, such as diet and gut microbiota composition, play significant roles in the development of IBD and the recurrence of intestinal inflammation (2). Despite this, the etiology and regulatory mechanisms of IBD remain poorly understood, and new, more effective therapies are urgently needed for patients who do not respond to current treatments (3, 4).

Taste receptors and their homologs are G protein-coupled receptors (GPCRs) that detect sweet, umami, and bitter compounds (5). They were first identified on the tongue and are considered molecular sensors that evaluate the composition of food in the oral cavity (6). Recent evidence has shown that taste signal transduction pathways also occur outside the tongue (7, 8), though the specific functions of these extra-oral taste receptors remain largely unknown.

The Taste 2 receptor family (Tas2r) is responsible for bitter taste transduction and has also been reported to regulate endocrine, behavioral, and immune responses (9). This family consists of approximately 35 functional members in rodents and 25 in humans (9, 10). Growing evidence indicates that Tas2rs are widely expressed throughout the body, where they mediate various non-tasting functions. For example, in the airway, Tas2r activation can increase ciliary motility, inhibit smooth muscle contraction, and participate in the innate immune response in airway epithelial cells (11, 12). Tas2rs have also been identified in immune cells. Tas2r138 is expressed in mouse neutrophils, where it protects against *Pseudomonas aeruginosa* infection by promoting AHL-12 production (13). Furthermore, human Tas2r14 can enhance macrophage phagocytosis through nitric oxide production (14). Recently, Tas2r126, Tas2r135, and Tas2r143 have been shown to be expressed in mouse neutrophils but not in macrophages or T and B lymphocytes, and these receptors regulate neutrophil migration through the ROCK-MLC2 signaling pathway (15).

Studies have also found that Tas2rs are expressed in the gut, where they can evoke Ca^2+^ signaling in enteroendocrine STC-1 cells, although they fail to trigger Ca^2+^ signaling in cell lines that do not express Tas2rs (16). Tas2rs help defend the body against infection-causing pathogens and maintain microbiota balance (17). Tuft cells, a rare type of intestinal epithelial cell, promote type 2 immunity in response to intestinal parasitic infection (18, 19). While several taste-signaling proteins have been identified in tuft cells, their precise functions remain unclear, although they may mediate type 2 immune responses in the gut. Another study showed that the loss of α-gustducin, a key component of taste GPCR signaling, leads to aggravated colitis in mice, with increased levels of tumor necrosis factor (TNF) and gamma interferon (IFN-γ) in the colon (20). These findings suggest that α-gustducin, together with its specific ligand, Tas2rs, plays an important role in the gut mucosal immune response.

Tas2r105, a specific member of the Tas2r family, was reported to be widely expressed in extraoral tissues and have important functions outside the gustatory system *in vivo* (21, 22). It is found in the renal corpuscle, where it helps maintain the structure of the glomerulus and renal tubules (21). Recent studies have revealed that periodontal Tas2r105 is coupled with gustducin and regulates host innate immunity by interacting with metabolites produced by oral bacteria (23). Given the role of Tas2rs and their downstream signaling components in immune response, we observed a significant increase in Tas2r105 expression in colitis mice. Based on this, we hypothesize that Tas2r105 may play an immune regulatory role in the pathogenesis of IBD. To further investigate this hypothesis, we conducted the following experimental study.

## MATERIALS AND METHODS

### Mice

All mice were of the C57BL/6 background. Twelve male wild-type (WT) mice (6–7 months old) were purchased from Slaccas company (Shanghai, China). Ten male Tas2r105 knockout mice (KO) (6–7 months old) were supplied by Shanghai Public Health Clinical Center. Tas2r105 KO mice were generated by Crispr/Cas9 technology as previously reported (24). They were housed in the specific pathogen-free (SPF) animal individually ventilated cages and maintained at 23 ± 1℃, 45%–65% humidity on a 12 h dark/light cycle. They have free access to food and water. All animal experiments were approved by the Institute Ethics Committee at Shanghai Public Health Clinical Center (2019-A030-01) and performed in accordance with national and EU guidelines.

### Colitis induction and evaluation of disease severity

All the mice have free access to food and water for a week. One week later, the mice were divided into four groups, WT-H2O (*n* = 6), WT-DSS (*n* = 6), Tas2r105 KO-H2O (*n* = 5), and Tas2r105 KO-DSS (*n* = 5). Acute colitis model was induced by giving mice 3.5% dextran sulfate sodium (DSS; MW 36,000–50,000, MP Biomedicals, America) in drinking water for 7 days. The control mice were given sterile water at the same time. Body weight and stool character were observed daily. Fecal occult blood tests were performed according to the instructions. The disease activity index (DAI) was calculated as described in Table S1. On day 8, the mice were anesthetized by intraperitoneal injection of pentobarbital. Peripheral blood, gut tissues, and the feces were collected for further investigation.

### Immunofluorescence and immunohistochemistry analysis

For immunohistochemistry (IHC) staining, parts of the colon were fixed in 4% paraformaldehyde (PFA) at 4°C overnight and then embedded in paraffin. The biopsies were cut into 3 μm-thick sections and stained with hematoxylin and eosin (H&E). Histological scoring of the colons was performed based on a combination of epithelial damage and inflammatory cell infiltration (Table S2). Individual scores were assigned as follows: 0 for no tissue damage and inflammation, 1 for focal damage and inflammation, 2 for patchy tissue damage and inflammation, and 3 for diffuse tissue damage and inflammation. The average scores for each group were calculated.

As for the immunofluorescence assay, the sections were incubated with the indicated primary antibodies overnight at 4°C and then with species-specific secondary antibodies for 1 h at room temperature. Rabbit anti-occludin mAb (GB111401, Servicebio, China) and rabbit anti-ZO-1 mAb (GB11195, Servicebio, China) were used to evaluate the integrity of the gut barrier. Mice anti-CD4 mAb (GB11064), anti-CD8 mAb (GB15068), anti-F4/80 mAb (GB113373), and anti-Ly6G mAb (GB11229) were applied to investigate the infiltration of immunocytes in the colon. All the above antibodies were purchased from the Servicebio company of China. The images were captured using a microscope (Olympus, Cat. #IX73). Occludin-positive and ZO-1-positive cells per crypt were calculated. The percentage of CD4, CD8, F4/80, and Ly6G positive cells was also detected.

### Quantitative real-time PCR (qRT-PCR) analysis

Small segments of the distal colon were dissolved by Trizol reagent to extract total RNA. cDNA was synthesized using a first-strand cDNA synthesis kit, and qRT-PCR was conducted using the QuantStudioTM6 Flex real-time PCR instrument (ABI, USA) with the SYBR Premix Ex TaqTM II mix (Takara, Japan). The primer sequences are listed in Table 1. The 2^−ΔΔCT^ method was used to calculate the relative mRNA levels normalized to the housekeeping gene glyceraldehyde-3-phosphate dehydrogenase (GAPDH).

**TABLE 1:** Primers used in qRT-PCR assay

Name	Sequence (5′−3′)
Mouse	
Occludin-F	CAGTTAGATGATGTGACCTCT
Occludin-R	GAGATACCTCAGTGTTACAAC
ZO-1-F	AGTCCCAGTGGAAGTGCTG
ZO-1-R	GTGCTAAGTCCTGCGAGGAT
IFN-γ-F	CAGCAACAGCAAGGCGAAA
IFN-γ-R	CTGGACCTGTGGGTTGTTGAC
TNF-α-F	CAGGCGGTGCCTATGTCTC
TNF-α-R	CGATCACCCCGAAGTTCAGTAG
GAPDH-F	ACTCCACTCACGGCAAATTC
GAPDH-R	TCTCCATGGTGGTGAAGACA
Gut flora of mouse	
16s V34-F	341F CCTAYGGGRBGCASCAG
16s V34-R	806R GGACTACNNGGGTATCTAAT

### Goblet cell number count and glycogen synthesis

The colons were fixed in 4% PFA at 4°C overnight and embedded in paraffin. The biopsies were then cut into 3 μm-thick sections. Goblet cells were stained using Alcian blue staining methods and periodic acid–Schiff (PAS) staining as previously described (25, 26).

### 16s rRNA gene sequencing of the gut microbiome

Total DNA was extracted from the stool samples from WT control and Tas2r105 KO control mice, and DNA concentration and purity were analyzed on agarose gels. The V34 region of the 16s rRNA gene was amplified on a PCR system, and NEB Next Ultra DNA Library Prep Kit (Illumina, San Diego, CA, USA Catalog#: E7370L) was used to generate sequencing libraries according to the manufacturer’s instructions. The corresponding primers used are listed in Table 1. Fastp (v0.22.0) and FLASH (v1.2.11) software were used to obtain clean tags data. The library was sequenced on the NovaSeq6000 system (Illumina, USA). UPARSE (v7.0.1001) algorithm was used to analyze the sequences. USEARCH (v7) was used to cluster all effective tags for all samples. Sequences with ≥97% similarity were assigned to the same operational taxonomic units (OTUs). According to the sequence data, the microbial composition and biodiversity were analyzed. Alpha diversity was applied to evaluate the complexity of species complexity within a sample through Chao1 and the Shannon’s index. Beta diversity was used to evaluate differences in species diversity between samples. Nonmetric multidimensional scaling (NMDS) analysis and principal coordinate analysis (PCoA) based on unweighted Unifrac were adopted to identify differences between groups. Linear discriminant analysis effect size (LEfSe) was applied to highlight the core bacterial phenotypes from the phylum to the genus contributing to the variations in the microbiota composition. LEfSe (v1.1.2) was used to conduct the LEfSe. And the statistical analysis was carried out by the RStudio program (v4.2.0). Besides, the relative abundance of the specific family and genus was further investigated. Phyloseq (v1.40.0) and Vega (v2.6.2) of RStudio were applied to process the alpha diversity, Chao1, and Shannon’s index. Phyloseq was applied to conduct PCoA and NMDS analysis.

### Gut metabolomics analysis

The gut metabolic difference between WT control and Tas2r105 KO control mice was evaluated by liquid chromatography–mass spectrometry (LC–MS) at Wuhan Metware Biotechnology Co, Ltd (Wuhan, China), the procedures of LC–MS were illustrated as described previously (27, 28). Quality control sample (QC) is prepared by mixing sample extracts to analyze the repeatability of the sample under the same treatment. In the process of analysis, a quality control sample is inserted into every 10 test samples to monitor the repeatability of the analysis process. The repeatability of metabolite extraction and detection can be judged through overlapping display analysis of total ion flow graphs (TIC graphs). The high stability of the instrument provides an important guarantee for the repeatability and reliability of our data. Analyst 1.6.3 software was used to process the mass spectrum data. Metabolite data were log2-transformed for statistical analysis. Metabolic identification information was obtained by searching the laboratory’s self-built database. Different metabolites were screened combining through fold change and variable importance in projection (VIP) value.

The multivariate statistical analysis includes principal component analysis (PCA) and orthogonal partial least-squares discriminant analysis (OPLS-DA). Differential metabolites were screened, and the criteria were as follows: variable importance in projection (VIP) scores ≥ 1; *P* < 0.05; fold change ≥ 2 or ≤ 0.5. Volcano plots and heatmaps were used to illustrate the differences in metabolites between groups. The KEGG database (http://www.kegg.jp/kegg/pathway.html) was used to perform pathway enrichment analysis of metabolites in difference.

### Statistical analysis

All data were presented as mean ± standard deviation (SD). One-way analysis of variance (ANOVA) was used to analyze the difference between the four groups. If the variance is homogeneous (*P* > 0.05), the least significant difference (LSD) method is used for post hoc testing; otherwise (*P* < 0.05), Dunnett’s T3 test is applied for the post-ANOVA analysis. The percentage of the infiltrated immunocytes between WT-DSS and KO-DSS was analyzed by *t*-test. TNF-α/IFN-γ gene expression between WT mice and KO mice, KO-H2O and KO-DSS, and WT-DSS and KO-DSS were also analyzed by *t*-test. Spearman correlation tests were applied to identify the correlation between gut microbiome and metabolome. The graphs were constructed by R software (v4.2.0) and GraphPad Prism 8. *P* values < 0.05 were considered to be statistically significant.

## RESULTS

### Mice deficient in Tas2r105 are highly susceptible to DSS-induced colitis

**Fig 1:**
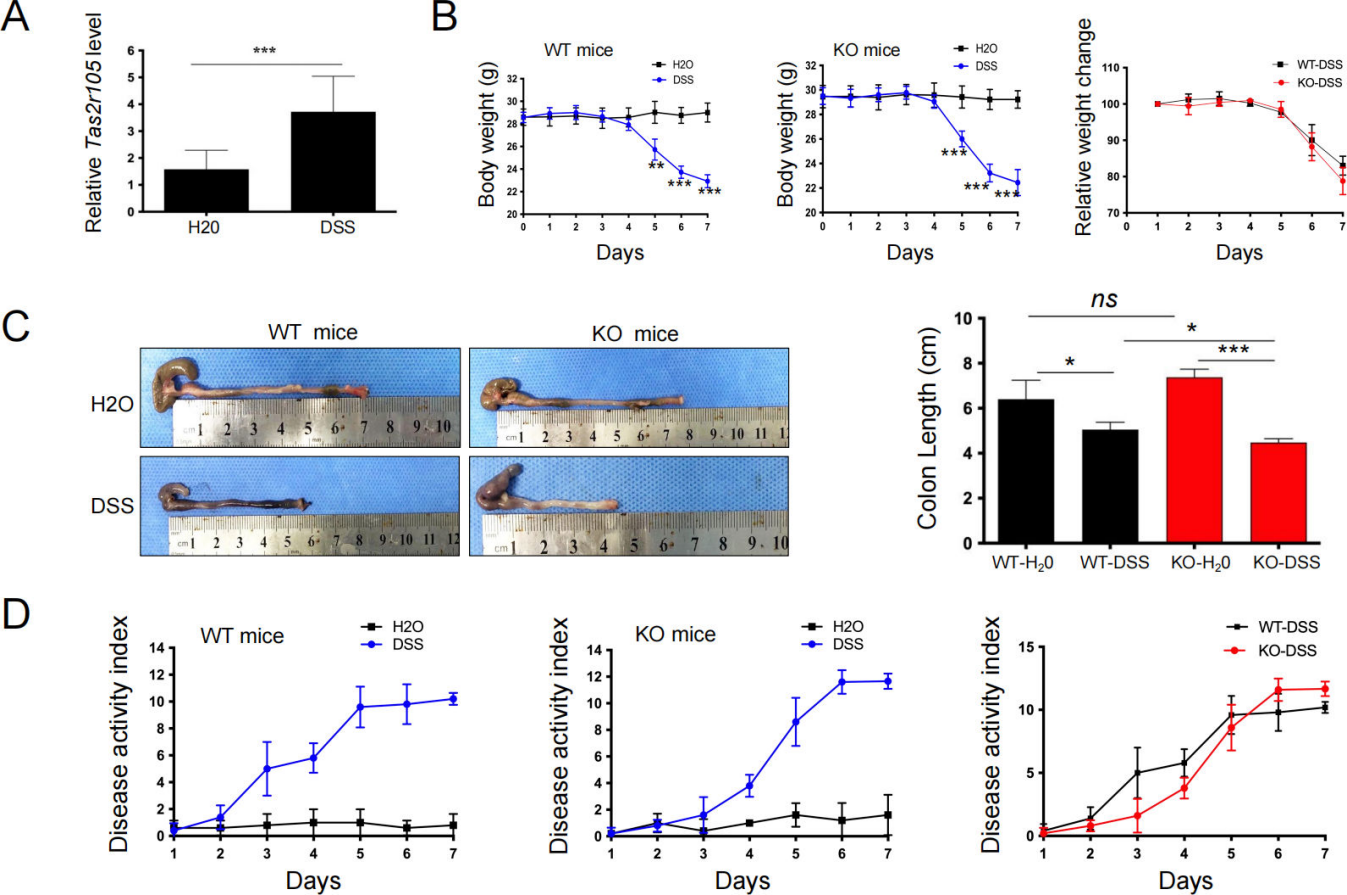
Tas2r105 KO mice are more susceptible to DSS-induced colitis. (A) *Tas2r105* is increased in the inflamed colon. (B) Comparison of body weight change between the Tas2r105 KO mice and the WT mice. (C) Comparison of colon length between the four groups of mice. (D) Comparison of disease activity index between different groups of mice. All data are shown as mean ± SD (*P < 0.05, **P < 0.01, ***P < 0.001, *ns:* non-significant).

**Fig 2:**
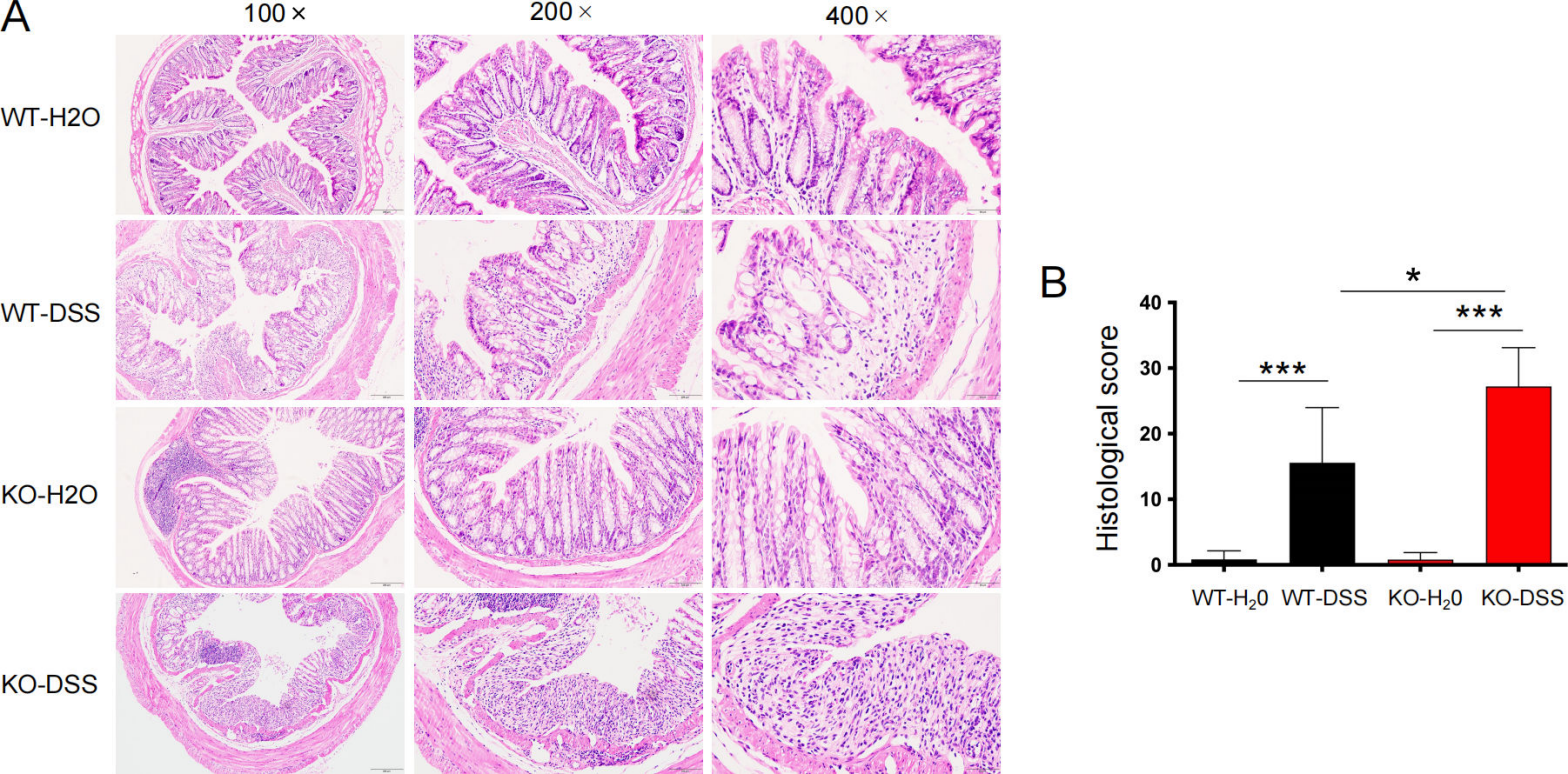
The intestinal mucosa of Tas2r105 KO mice is more seriously damaged after DSS drinking. (A) Representative images of H&E staining of the colon tissues. (B) The KO-DSS mice showed increased histological score compared with the WT-DSS mice. All data are shown as mean ± SD (*P < 0.05, ***P < 0.001).

Increased Tas2r105 gene expression was detected in the inflamed colons of mice (Fig. 1A). To determine whether Tas2r105 pathways are involved in the immune response to colitis, we tested Tas2r105 knockout (KO) mice in the well-established DSS-induced acute colitis model. After seven consecutive days of DSS drinking, both WT and KO mice showed significant body weight loss, but no significant difference was observed between the two groups (Fig. 1B). Interestingly, the colon length increased in Tas2r105 KO mice, although the difference was not statistically significant compared with the WT mice. However, in the colitis model, the KO mice exhibited a much shorter colon length than the WT mice (Fig. 1C), suggesting more severe colitis in the Tas2r105 KO mice. The DAI score, which is based on the severity of diarrhea and rectal bleeding, increased sharply in the KO-DSS mice and surpassed the WT-DSS group on day 5 (Fig. 1D). Further histopathological analysis revealed more severe colon damage in the KO mice compared with the WT mice following DSS administration (Fig. 2A). Tas2r105 KO mice had significantly more crypt loss and immune cell infiltration. Histological scoring showed higher tissue injury scores in DSS-treated KO mice compared with the DSS-treated WT mice (Fig. 2B). These results indicate that Tas2r105 plays a crucial role in protecting mice from DSS-induced colon damage.

### Tas2r105 KO mice exhibit aggravated intestinal barrier destruction and goblet cell loss in the colitis model

**Fig 3:**
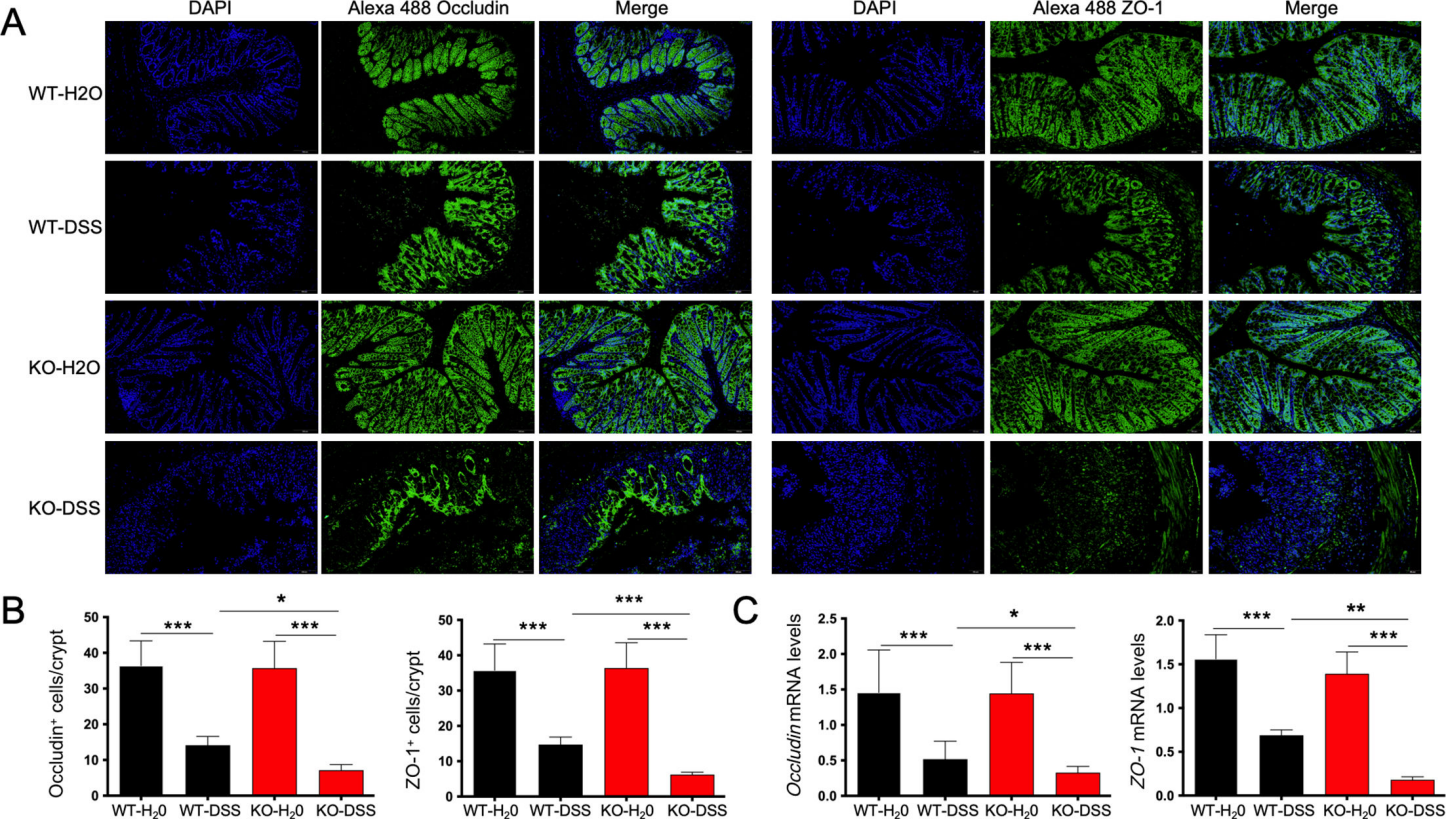
The gut barrier of Tas2r105 KO mice is severely destroyed by DSS treatment. (A) Typical immunofluorescence pictures of occludin and ZO-1 expression in the colon for the Tas2r105 KO mice and the WT mice. The nucleus was blue stained by DAPI, while the target proteins were green stained by Alexa 488, the scale bar was labeled on each image. (B) Statistical figures of the occludin^+^ and ZO-1^+^ cells per crypt between the four groups of mice. (C) RT-PCR assay to detect occludin and ZO-1 mRNA levels in the colon. All data are shown as mean ± SD (*P < 0.05, **P < 0.01, ***P < 0.001).

**Fig 4:**
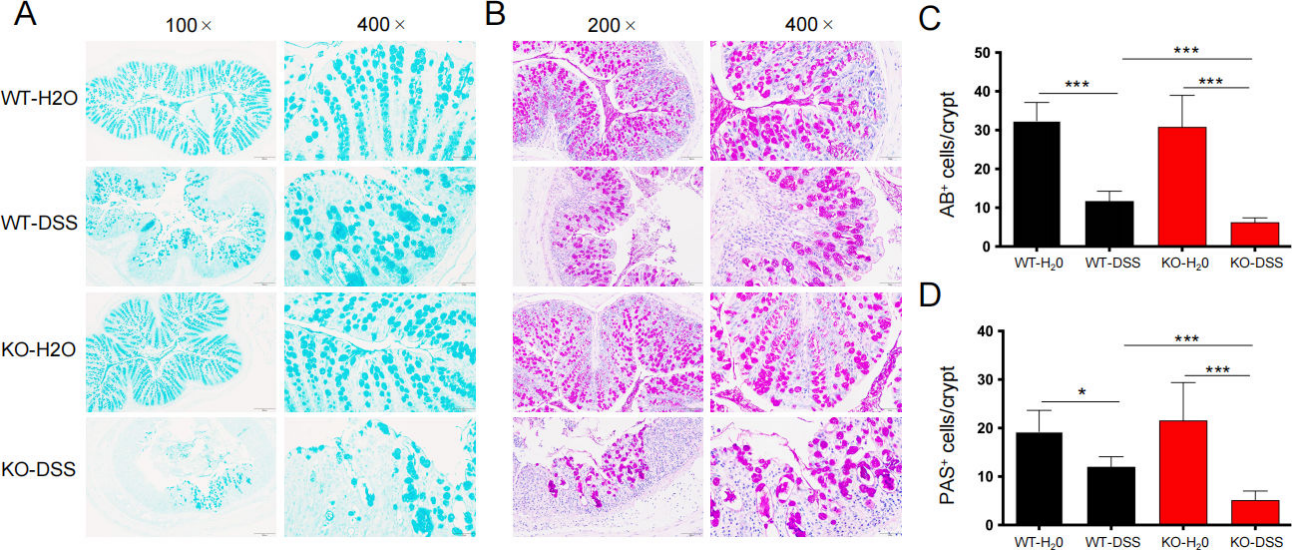
The proportion of goblet cells was downregulated when Tas2r105 was knocked out. (A) Representative images of AB and PAS staining (B) of the colon tissues for the four groups of mice, the scale bar was labeled on each image. (C) The statistical figures of the AB^+^ cells and PAS^+^ cells (D) per crypt. All data are shown as mean ± SD (*P < 0.05, ***P < 0.001).

Intestinal barrier destruction and the resulting increased intestinal permeability have been hypothesized as risk factors for IBD. In the intestine, tight junctions (TJs) play a central role in regulating the gut barrier. Occludin and zonula occludens-1 (ZO-1) are two key components of TJs, and their removal or reduced expression leads to barrier dysfunction. We investigated the expression of TJ proteins in KO-H2O mice and found no significant difference compared with the WT-H2O mice. However, in the DSS-treated mice, there was significant downregulation of occludin and ZO-1 protein levels in KO mice compared with WT mice, and the result was further confirmed at the mRNA level (Fig. 3A through C). The intestinal epithelium forms a physical barrier that is further protected by a mucus layer, which helps maintain intestinal health. Given the fact that mucus is produced by goblet cells, we investigated the number of goblet cells in the colon by AB/PAS staining. Compared with the WT-DSS mice, the KO-DSS mice exhibited fewer goblet cells and a significantly thinner mucus layer in the gut (Fig. 4A through D). These findings suggest that the colon of Tas2r105 KO mice is more vulnerable to DSS-induced injury, indicating that Tas2r105 may play a protective role in intestinal inflammation.

### More macrophages and fewer T lymphocytes infiltrate the lamina propria of Tas2r105 KO mice

**Fig 5:**
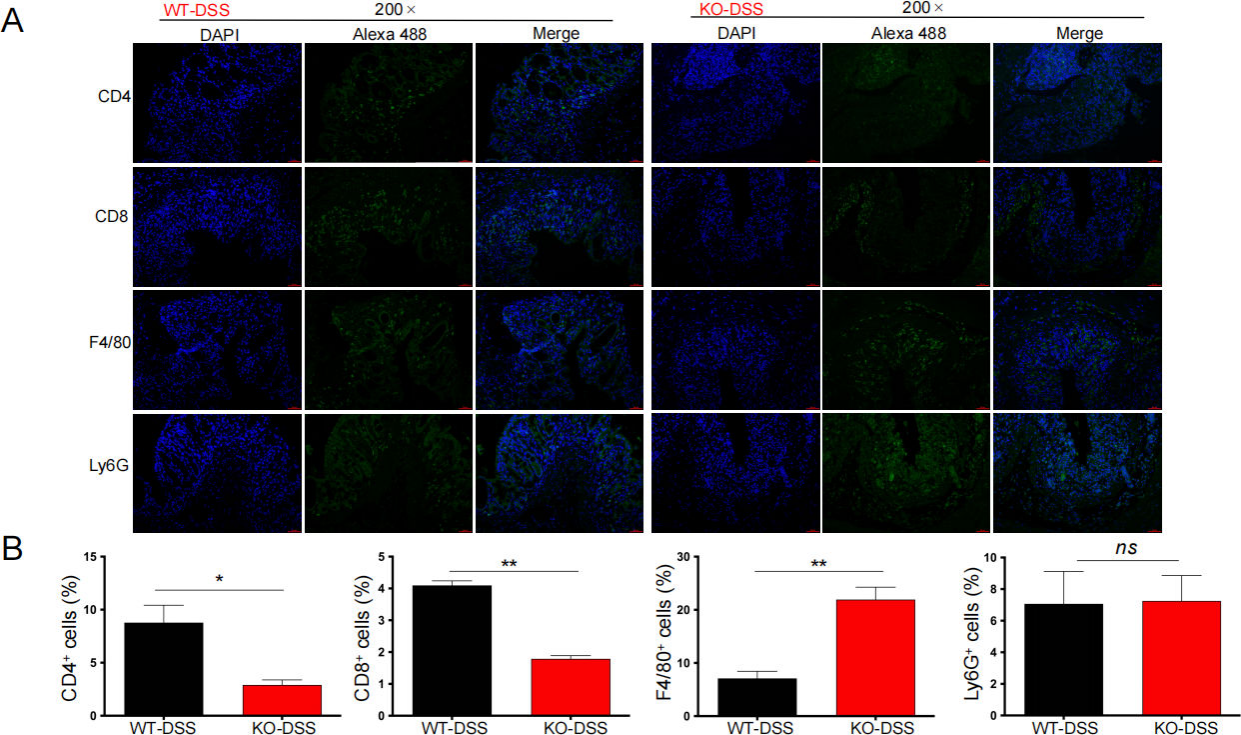
More macrophages were recruited into the intestinal mucosa when the Tas2r105 gene was absent. (A) Representative immunofluorescence images of CD4^+^T, CD8^+^T, F4/80^+^ macrophages, and Ly6G^+^ neutrophils saturated in the gut mucosa. Nuclei were blue stained by DAPI, while the target cells were green stained by Alexa 488; the scale bar was labeled on each image. (B) The statistical figures of CD4^+^T, CD8^+^T, F4/80^+^ macrophages, and Ly6G^+^ neutrophils per crypt between the WT-DSS and KO-DSS mice. All data are shown as mean ± SD (*P < 0.05, **P < 0.01, *ns:* non-significant).

**Fig 6:**
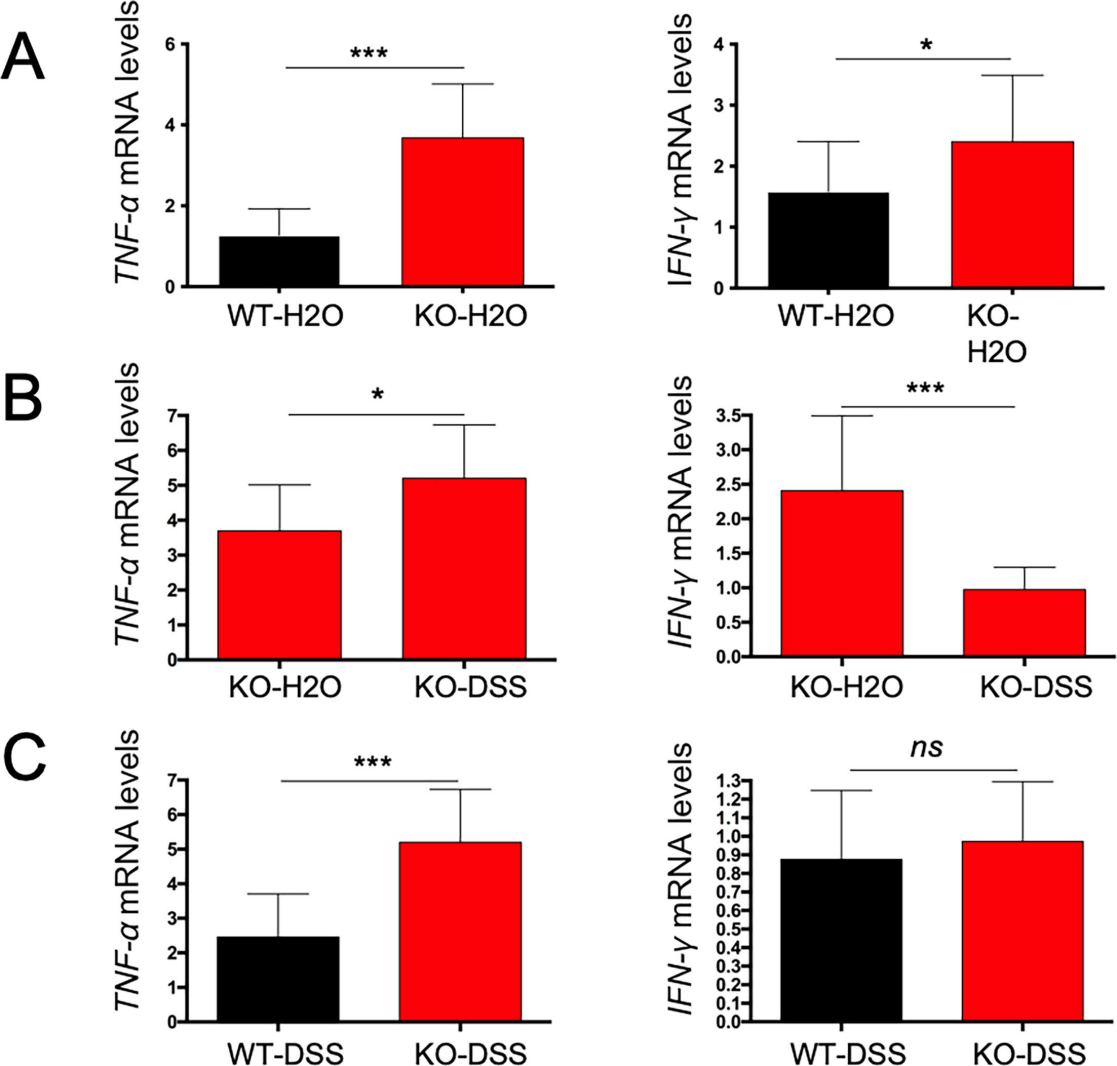
The expression of proinflammatory factors was different between Tas2r105 KO mice and the WT mice. (A) RT-PCR assay to detect TNF-α and IFN-γ mRNA levels for the WT-H2O and KO-H2O mice. (B) RT-PCR to detect TNF-α and IFN-γ mRNA levels for the Tas2r105 KO mice before and after DSS treatment. (C) RT-PCR to detect TNF-α and IFN-γ expression between the Tas2r105 KO mice and WT mice after DSS treatment. All data are shown as mean ± SD (*P < 0.05, ***P < 0.001, *ns:* non-significant).

Our results show that Tas2r105 KO mice are more susceptible to DSS-induced injury, suggesting that Tas2r105 might act as an anti-inflammatory molecule. To test this hypothesis, we performed immunofluorescence assays to evaluate immune cell infiltration in the gut mucosa. We used F4/80 and Ly6G antibodies to distinguish macrophages and neutrophils, respectively. In the DSS-treated groups, we observed a reduction in CD4^+^ T and CD8^+^ T lymphocytes in the KO mice. In contrast, more F4/80^+^ macrophages were present in the lamina propria mucosa, with no significant difference in neutrophil infiltration (Fig. 5A and B). Furthermore, intestinal mRNA levels for TNF-α and IFN-γ were sharply increased in the KO-H2O mice compared with those of WT-H2O mice (Fig. 6A). Notably, TNF-α expression was elevated in the KO mice upon DSS treatment, while IFN-γ levels were downregulated (Fig. 6B). Compared with DSS-treated WT mice, DSS-treated KO mice had increased TNF-α levels, with no change in IFN-γ expression (Fig. 6C). These results suggest that Tas2r105 may protect the intestine from inflammation, primarily by suppressing the TNF-α pathway.

### The gut microbiota is structurally and functionally altered in Tas2r105 KO mice

It is well established that gut flora and their metabolites play a critical role in IBD pathogenesis. One of the newly revealed functions of taste receptors is modulation of immune responses to microbes and metabolism (13, 23). Since taste receptors influence food perception and dietary habits, we hypothesize that Tas2r105 may regulate the gut microbiota, which could, in turn, affect colonic injury in Tas2r105 KO mice. To confirm this hypothesis, we performed 16s rRNA gene sequencing and metabolomic analysis to assess microbial alterations and metabolite changes in stool samples of WT control mice and KO control mice. This was done to evaluate the effect of the Tas2r105 KO in the absence of colitis. Additionally, a combined analysis of the microbiome and metabolomics was used to explore potential mechanisms.

**Fig 7:**
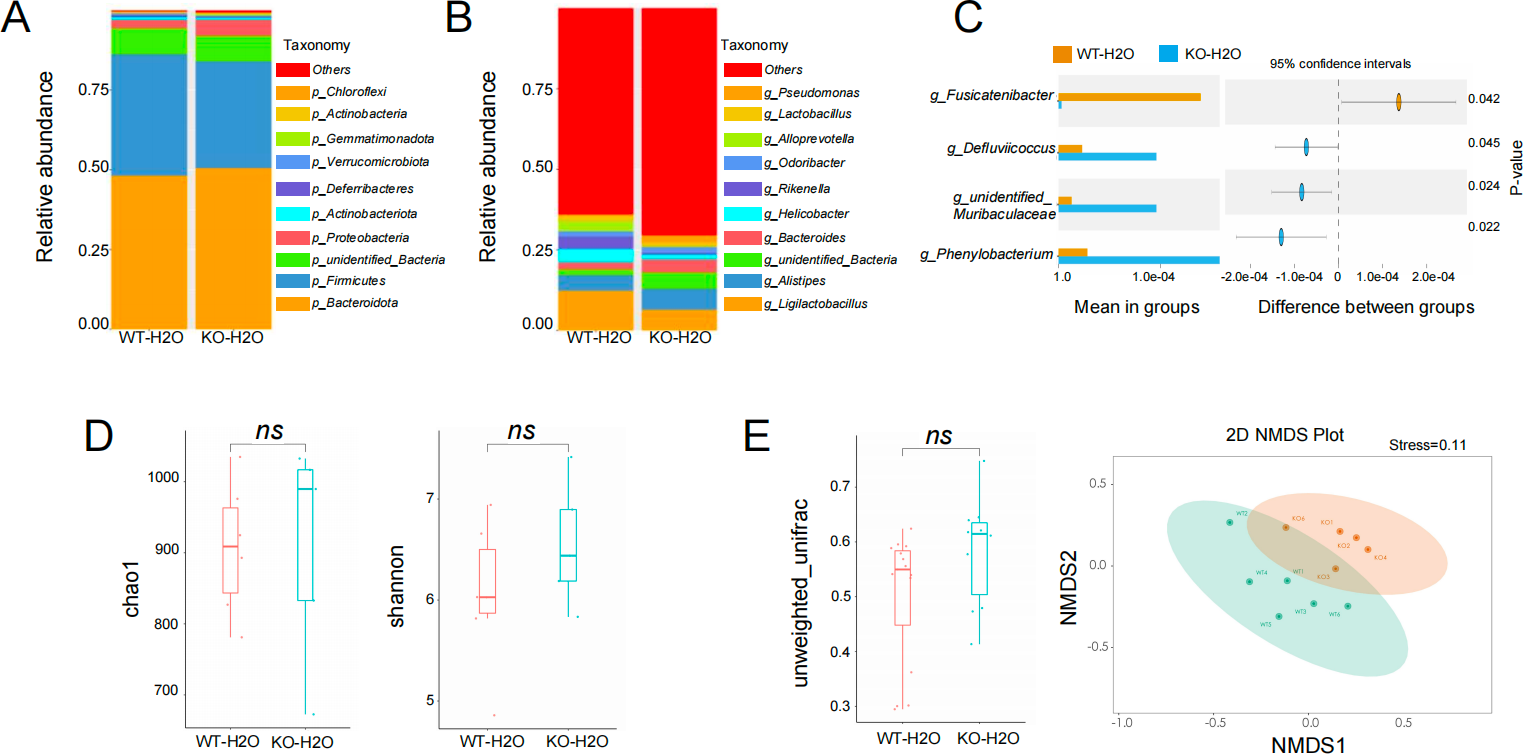
Altered gut microbiota in Tas2r105 KO mice. (A) The alpha diversity between the WT-H2O and KO-H2O mice in the phylum and genus level (B). (C) Statistical analysis of the gut microbiota in WT-H2O (orange) and KO-H2O (blue) groups by Student’s *t*-test. (D) Chao1 and Shannon indexes were applied to assess the richness and diversity of the gut microbiome for the two groups of mice. (E) Beta diversity was measured by NMDS analysis based on the OTU levels (*ns:* non-significant).

**Fig 8:**
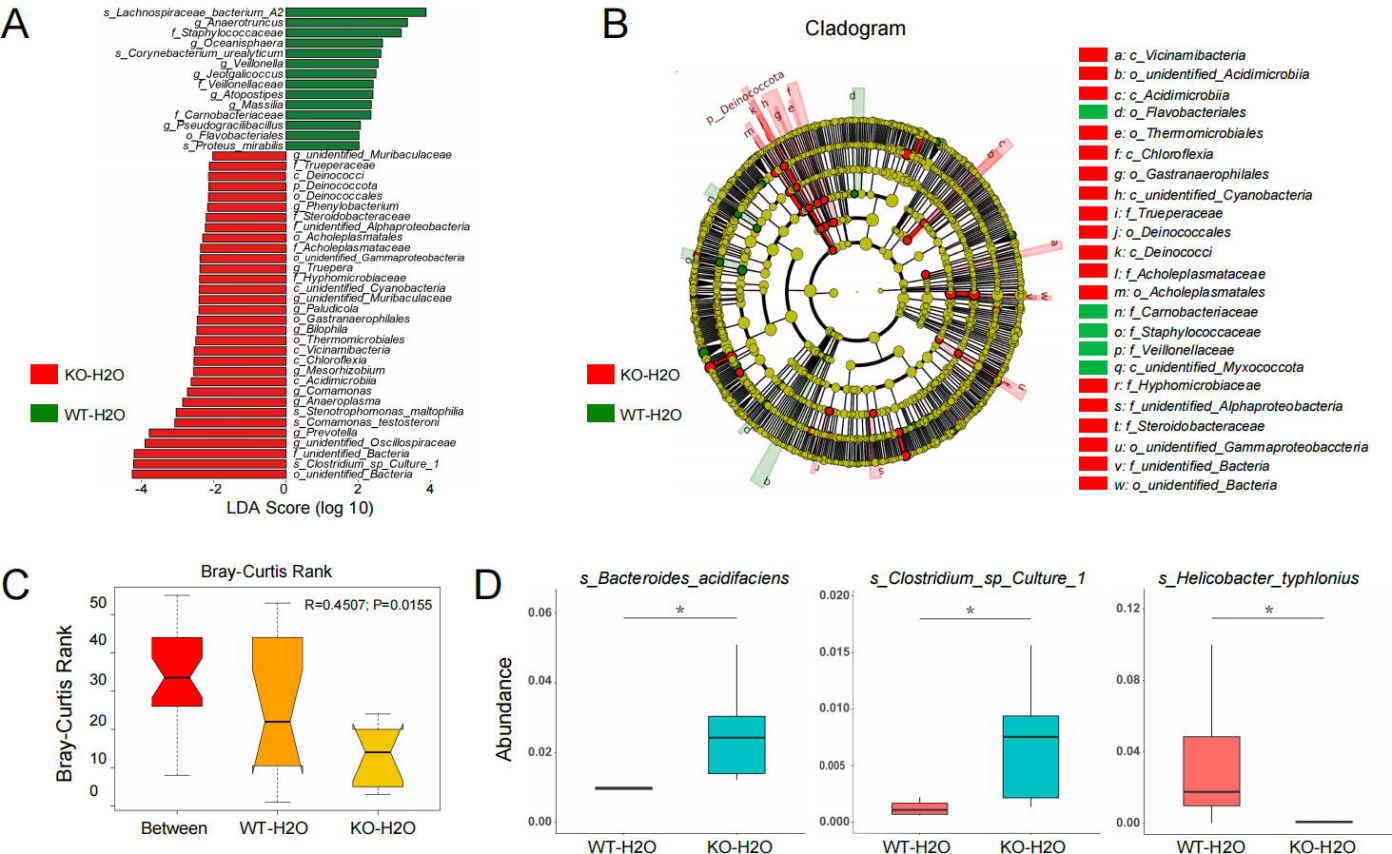
Microbial community dynamics of the gut when the Tas2r105 gene was absent. (A) LDA coupled with effect size measurements in the WT-H2O (green) and KO-H2O (red) mice. Enriched taxa in the two groups are displayed with linear discriminant analysis (LDA) scores beyond the threshold of 4.0. (B) Cladogram representation of the gut microbiota in the WT-H2O versus the KO-H2O group by 16s rRNA sequencing. Enriched taxa in the WT-H2O (green) and KO-H2O (red) groups are indicated. The brightness of each dot is correlated with its LDA effect size. (C) ANOSIM analysis results. “Between” represents the difference between groups; the greater the distance is, the greater the difference is. The thickness is the sample size. (D) Significant taxonomic differences between groups (species level) (*P < 0.05).

16s rRNA sequencing revealed a total of 2,196 operational taxonomic units (OTUs). At the phylum level, the two dominant bacterial phyla, *Bacteroidota* and *Firmicutes*, were present in both groups, but *Proteobacteria* levels were significantly higher, and *Firmicutes* were dramatically reduced in Tas2r105 KO mice (Fig. 7A). At the genus level, *Fusicatenibacter* was significantly downregulated (*P* = 0.042), while *Defluviicoccus* (*P* = 0.045) and *Phenylobacterium* (*P* = 0.022) were increased in the KO mice (Fig. 7B and C). Microbial richness and diversity were assessed using Chao1 and Shannon indexes, but no significant differences were found between the two groups (Fig. 7D), indicating that Tas2r105 deletion had little effect on the α-diversity of the gut microbiota. NMDS analysis, based on OTU levels, demonstrated that samples from the same group clustered together, suggesting similar bacterial compositions within each group (stress value = 0.11). No significant difference in β-diversity was observed between groups. The group-related difference was represented by PC1, which explained 25.91% of the total variation in microbiota composition (Fig. 7E). Additionally, LEfSe was used to highlight bacterial phenotypes contributing to microbiota variations, with a threshold of 4. Figure 8A and B show that the WT group was enriched with *Lachnospiraceae bacterium A2*, *Anaerotruncus*, and the *Staphylococcaceae* family, while the KO mice were enriched with *Clostridium sp* culture 1, *Comamonadaceae*, and *Oscillospiraceae*. Furthermore, analysis of similarities (ANOSIM) analysis revealed a significant difference in microbiota structure between the two groups (*R* = 0.45, *P* = 0.015) (Fig. 8C). At the species level, there was a marked increase in *Bacteroides acidifaciens* and *Clostridium* sp. culture 1, alongside a significant reduction in *Helicobacter typhlonius* (Fig. 8D).

### Tas2r105 deletion modulates fecal metabolites

**Fig 9:**
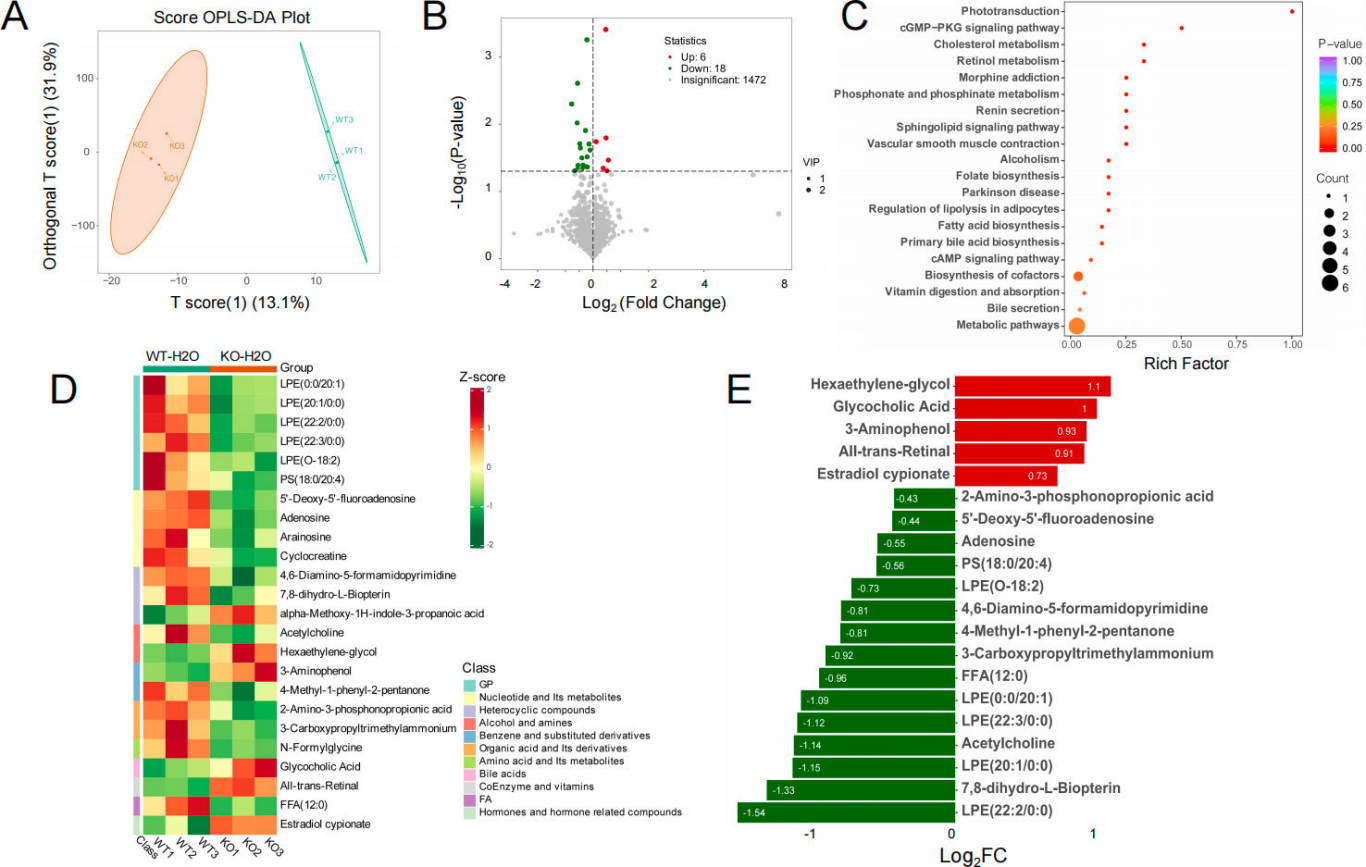
The Tas2r105 gene deletion significantly changed the host metabolic profile. (A) Score OPLS-DA plot to reveal the different fecal metabolites between the WT-H2O (green) and KO-H2O (orange) groups. Metabolites with VIP ≥1.0; *P* < 0.05 were defined as differential metabolites. (B) Volcano plot showing differential metabolites between the WT-H2O and KO-H2O groups. (C) KEGG enrichment analysis of metabolites between WT-H2O and KO-H2O groups. The ordinate represents the distinct KEGG pathways, and the abscissa represents the rich factor. The color of the dots represents the *P* values of enrichment. The size of the dots represents the gene number of enrichments. (D) Heatmap and the cluster analysis of serum metabolites between the WT-H2O and KO-H2O groups. Each column represents a sample, and each row stands for a metabolite. (E) Bar chart to show the top 20 differential metabolites between the WT-H2O and KO-H2O mice.

The gut metabolomic profiles were determined by LC–MS. An OPLS-DA model was adopted to further analyze the differential metabolites between the two groups. The OPLS-DA score indicated that the two groups were separated into different regions (Fig. 9A). Based on the results of OPLS-DA, we use VIP as a threshold to further screen differential metabolites. Metabolites with VIP ≥1.0 and *P* < 0.05 were defined as different metabolites, and finally, 24 differential metabolites were detected between the WT-H2O versus KO-H2O group, among which, six metabolites increased significantly, while 18 metabolites decreased compared with the WT mice (Fig. 9B). The detailed information of the 24 metabolites is listed in Tables S3 and S4, and the multiple reaction monitoring (MRM) scans for the 24 metabolites were listed in Fig. S1. As shown in Fig. 9C, the relative content of glycerophospholipids (GPs) and nucleotide-associated metabolites was decreased significantly in mice feces after the Tas2r105 gene was deleted. The KO mice were rich with hexaethylene-glycol, glycocholic acid, 3-aminophenol, and all trans-retinal, while the WT mice were rich with lysophosphatidylethanolamine (LPE), acetylcholine, adenosine, 5′-deoxy-5′-fluoroadenosine, and free fatty acid (FFA) (Fig. 9D and E).

### Correlations between the gut microbiota and metabolites in Tas2r105 KO mice

**Fig 10:**
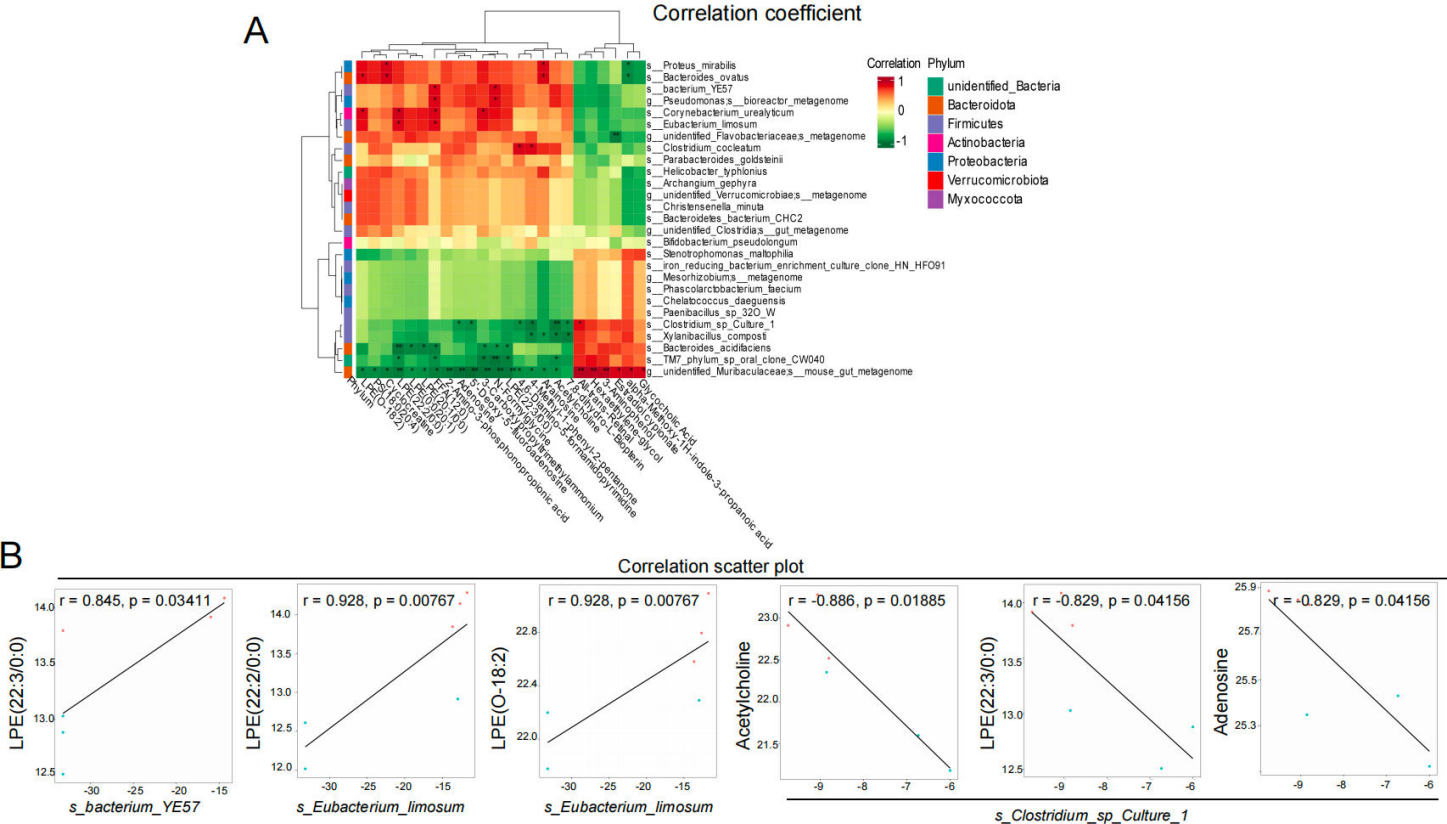
There is a significant correlation between the different microbiota and metabolites in the feces for the Tas2r105 KO and WT water-drinking mice. (A) Pearson correlation analysis between the relative abundance of microorganism and metabolites in the WT-H2O and KO-H2O groups. The correlation effect is indicated by a color gradient from green (negative correlation) to red (positive correlation). (B) Scatter plots illustrating the statistical correlation between the relative abundance of altered gut bacterial species and LC–MS spectrum intensities of the differential metabolites (*P < 0.05, **P < 0.01).

Spearman’s correlation coefficient was calculated to explore the potential relationships between the gut microbiome changes and metabolic products between the two groups (Fig. 10A). The correlations with *r* ≥ 0.8 and *P* < 0.05 were considered significant. The results of the correlation analysis were listed in Table S5. The results showed that the unidentified *Muribaculaceae* was negatively correlated with LPE, FFA, 2-amino-3-phosphonopropionic acid, adenosine, 5′-deoxy-5′-fluoroadenosine, 3-carboxypropyltrimethylammonium, N-formylglycine, arainosine, and acetylcholine. *Bacteroides acidifaciens* had a negative correlation with LPE, FFA, 3-carboxypropyltrimethylammonium, and N-formylglycine. *Clostridium* was negatively correlated with adenosine, 5′-deoxy-5′-fluoroadenosine, acetylcholine, and positively correlated with all trans-retinal. *Corynebacterium_urealyticum* was positively related to LPE, FFA, and 3-carboxypropyltrimethylammonium.

## DISCUSSION

Our senses of taste, sight, hearing, and smell allow us to consciously perceive the external environment. Taste sensing is a sophisticated process. Previously, Tas2rs were considered to be exclusively located on the tongue, where they initiate a signaling pathway that communicates bitter information to the brain (6, 29). However, recent studies have linked Tas2rs to several diseases affecting other systems. In humans, Tas2rs are expressed in cord blood-derived mast cells (CBMCs) and the mast cell line HMC1.2. Tas2r agonists have been shown to significantly inhibit histamine and PGD2 release from primary human mast cells (30).

In the present study, we observed increased Tas2r105 expression in the inflamed colon of mice with DSS-induced colitis, and we demonstrated that Tas2r105 deficiency exacerbated colitis by promoting the release of pro-inflammatory cytokines and aggravating epithelial barrier damage. Further microbiological and metabolomic studies suggested that the gut microbiota composition in Tas2r105 KO mice was altered, with a reduction in *Firmicutes* and an increased proportion of *Proteobacteria* and *Bacteroidetes*.

The intestinal barrier is primarily composed of mucus and epithelial cells. One of the most important functions of the intestinal epithelium is to absorb nutrients and form a critical barrier between the host and its microbiota (31). Among the key components of the intestinal barrier, tight junctions, which include claudins, occludins, ZO-1, and junction adhesion molecules, are crucial for maintaining the physical barrier of the intestine (32). Dysfunction of the mucosal barrier is associated with increased gut permeability and the development of various gastrointestinal diseases. Occludin and ZO-1 are two major components of TJs. In our study, we found downregulation of occludin and ZO-1 in the colonic epithelium of Tas2r105 KO mice treated with DSS, while no differences were observed in the water-treated groups. These results demonstrate that Tas2r105 deletion does not directly affect the transcription of occludin and ZO-1, but it renders the intestinal epithelium more vulnerable to DSS-induced damage. It is possible that Tas2r105 influences intestinal permeability through other mechanisms, which warrant further investigation in future studies.

IBD is a relapsing inflammatory disease in which immune cells, including dendritic cells, macrophages, T cells, and natural killer T cells, play a crucial role in disease pathogenesis. Given that inflammatory responses are a key indicator of colitis severity, we measured intestinal pro-inflammatory factors in our study. We found that the colon of Tas2r105 KO mice became significantly longer, and the KO-H2O mice had higher levels of IFN-γ and TNF-α in the colon compared with the WT-H2O mice. However, histological analysis revealed no significant mucosal damage in the KO-H2O mice. Immunofluorescence assays demonstrated that more F4/80^+^ macrophages infiltrated to the lamina propria (LP) of the colon, while fewer T cells were detected in the DSS-treated KO mice. Additionally, Tas2r105 KO mice exposed to DSS showed increased levels of pro-inflammatory cytokines, such as TNF-α, in colon tissues compared with WT-DSS mice. Based on these findings, we speculate that Tas2r105 might protect the colon from inflammatory injury partially through restricting the infiltration of macrophages to damaged colon.

Commensal bacteria and the immune system work in concert to maintain intestinal barrier homeostasis. A healthy gut microbiota benefits the host by aiding nutrient metabolism, preventing pathogen colonization, and maintaining epithelial integrity. Dysbiosis, or changes in gut flora, followed by the disruption of host–microbial mutualism, is thought to be a key event in the development of IBD (33, 34). Alterations in the gut microbiota have also been associated with disease activity and severity. In IBD, the relative proportions of *Firmicutes*, *Bacteroidetes*, *Proteobacteria*, and *Actinobacteria* are often altered, with a depletion of *Firmicutes* and *Bacteroidetes*, and an enrichment of *Proteobacteria* and *Actinobacteria* (35). Specifically, an expansion of *Proteobacteria* is thought to promote pro-inflammatory changes in IBD (36, 37). *Firmicutes*, one of the most abundant commensal bacteria in the colon, has been shown to exhibit anti-inflammatory effects and alleviate the progression of IBD (38). Its abundance is reduced in both CD and UC feces and is considered a good indicator of disease activity and severity. In CD patients with aggressive disease, the abundance of *Firmicutes* is significantly lower than in patients with non-aggressive disease (39), and similar findings have been reported for UC patients. *Proteobacteria* and *Bacteroidetes* are known to contribute to chronic inflammation and disrupt the intestinal barrier. As LPS-producing bacteria, *Proteobacteria* can trigger oxidative stress, ultimately leading to colon inflammation. In our study, we detected a dramatic reduction in *Firmicutes* and an increase in *Proteobacteria* in Tas2r105 KO mice. Additionally, we found that the abundance of *Stenotrophomonas*, *Comamonas*, and *Mesorhizobium*, all belonging to the *Proteobacteria* phylum, was significantly higher in KO mice. These findings are consistent with previous studies. The changes in gut flora help explain the increased colon inflammation observed in Tas2r105 KO mice. One possible mechanism is that the absence of Tas2r105 alters the host’s food perception, leading to changes in the gut microbiota.

Lysophospholipids (LPLs), which include lysophosphatidic acid (LPA), lysophosphatidylserine (LPS), lysophosphatidylcholine (LPC), lysophosphatidylglycerol (LPG), and LPE, were once thought to be metabolic byproducts or intermediates in phospholipid synthesis. However, recent studies have shown that LPLs serve as important signaling molecules, playing roles in tumorigenesis, neurodevelopment, immunity, and angiogenesis (40). LPE is a glycerophospholipid widely found in human organs and involved in lipid metabolism. In our study, we observed significantly lower levels of the differential metabolites LPE (0:0), LPE (20:1), LPE (22:2), LPE (22:3), LPE (O-18:2), and PS (18:0) in the Tas2r105 KO mice. LPE is an important modulator in the body. For example, berberine (BBR), an isoquinoline alkaloid isolated from Rhizoma coptis, is commonly used to treat hyperlipidemia in China, and metabolites including LPE (P-20:0_22:6), LPE (P-20:0_18:1), and LPE (P-19:0_20:4) are biomarkers of BBR regulation (41). Additionally, LPE has anti-inflammatory properties. Oral administration of LPE has been shown to attenuate peritonitis by reducing pro-inflammatory mediators (IL-1β, IL-6, and TNF-α) and increasing IL-10 secretion (42). Recently, LPE has been reported to regulate RORγt activity in Th17 cells and is essential for IL-17a transcription (43). Another study indicated that a mutant *Escherichia coli* strain with a single nucleotide polymorphism in the bacterial lipocalin gene was highly enriched in the feces of IBD patients, contributing to intestinal barrier disruption by reducing LPE production (44). These findings, together with our results, suggest that phospholipids, particularly LPE, are implicated in the pathogenesis of IBD and may provide a basis for new therapeutic strategies for colitis.

In conclusion, we have investigated the expression and function of bitter taste receptors in colonic colitis. Our findings suggest that Tas2r105 may help ameliorate gut inflammation by altering the host’s diet perception, thus affecting the gut microbiota and its metabolites. Our work highlights the important role of Tas2r105 as an anti-inflammatory gene in the development of IBD. Future studies should focus on elucidating the signaling pathways that mediate these biological effects in attenuating intestinal inflammation.

## SUPPLEMENTAL MATERIAL

Supplemental material msystems.01556-24-s0001.docx Supplemental tables and figure.

ASM does not own the copyrights to Supplemental Material that may be linked to, or accessed through, an article. The authors have granted ASM a non-exclusive, world-wide license to publish the Supplemental Material files. Please contact the corresponding author directly for reuse.
