# Supplementary material for: Tas2r105 ameliorates gut inflammation, possibly through influencing the gut microbiota and metabolites
Source: mSystems. 2025 Mar 13;10(4):e01556-24. doi: 10.1128/msystems.01556-24 (PMC12013267; doi:10.1128/msystems.01556-24)

**Supplementary Material Table 1 Disease activity index (DAI) scoring criteria**

| Score | Body weight loss (%) | Stool consistency | Rectal bleeding |
| --- | --- | --- | --- |
| 0 | None | Normal | Normal |
| 1 | 1-5 | N/A | N/A |
| 2 | 5-10 | Loose | Occult bleeding |
| 3 | 10-20 | N/A | N/A |
| 4 | >20 | Diarrhea | Gross bleeding |

**Supplementary Material Table 2 Scoring of severity of histological damage**

| Feature | Score | Description |
| --- | --- | --- |
| Severity of inflammation | 0 | None |
|  | 1 | Mild |
|  | 2 | Moderate |
|  | 3 | Severe |
|  |  |  |
| Extent of inflammation | 0 | None |
|  | 1 | Mucosa |
|  | 2 | Mucosa and submucosa |
|  | 3 | Transmural |
|  |  |  |
| Crypt Damage | 1 | 1/3 damaged |
|  | 2 | 2/3 damaged |
|  | 3 | Crypts lost, surface epithelium present |
|  | 4 | Crypts lost and surface epithelium lost |
|  |  |  |
| Percentage Involvement | 0 | 0% |
|  | 1 | 1-25% |
|  | 2 | 26-50% |
|  | 3 | 51-75% |
|  | 4 | 76-100% |

Scores were calculated by multiplying the score for the first three parameters by their percentage involvement, giving a maximum score of 40.

**Supplementary Material Table 3**

**Differential metabolites between the WT and Tas2r105 KO mice**

| No. | Index | Compounds | Class I | VIP | P-value | Log2FC | Type |
| --- | --- | --- | --- | --- | --- | --- | --- |
| 1 | MEDP2265 | 2-Amino-3-phosphonopropionic acid | Organic acid and Its derivatives | 2.4878 | 0.0306 | -0.4307 | down |
| 2 | MEDP2667 | 3-Aminophenol | Benzene and substituted derivatives | 2.5869 | 0.0160 | 0.9318 | up |
| 3 | MEDP1132 | 3-Carboxypropyltrimethylammonium | Organic acid and Its derivatives | 2.6159 | 0.0226 | -0.9158 | down |
| 4 | MEDN1509 | 4,6-Diamino-5-formamidopyrimidine | Heterocyclic compounds | 2.3590 | 0.0467 | -0.8069 | down |
| 5 | MEDP1732 | 4-Methyl-1-phenyl-2-pentanone | Benzene and substituted derivatives | 2.3518 | 0.0317 | -0.8123 | down |
| 6 | MEDP1202 | 5'-Deoxy-5'-fluoroadenosine | Nucleotide and Its metabolites | 2.7136 | 0.0006 | -0.4437 | down |
| 7 | MEDP0540 | 7,8-dihydro-L-Biopterin | Heterocyclic compounds | 2.1129 | 0.0493 | -1.3318 | down |
| 8 | MEDP1002 | Acetylcholine | Alcohol and amines | 2.3406 | 0.0473 | -1.1413 | down |
| 9 | MEDP0160 | Adenosine | Nucleotide and Its metabolites | 2.6382 | 0.0124 | -0.5478 | down |
| 10 | MEDP1499 | All-trans-Retinal | CoEnzyme and vitamins | 2.7179 | 0.0004 | 0.9149 | up |
| 11 | MEDP0390 | Arainosine | Nucleotide and Its metabolites | 2.3867 | 0.0433 | -0.4013 | down |
| 12 | MEDP2326 | Cyclocreatine | Nucleotide and Its metabolites | 2.4390 | 0.0243 | -0.2180 | down |
| 13 | MEDN2295 | Estradiol cypionate | Hormones and hormone related compounds | 2.3415 | 0.0451 | 0.7259 | up |
| 14 | MEDN0385 | FFA(12:0) | FA | 2.4800 | 0.0196 | -0.9616 | down |
| 15 | MEDN0112 | Glycocholic Acid | Bile acids | 2.3819 | 0.0494 | 1.0044 | up |
| 16 | MEDP2068 | Hexaethylene-glycol | Alcohol and amines | 2.6476 | 0.0342 | 1.1032 | up |
| 17 | MEDN2367 | LPE(0:0/20:1) | GP | 2.3609 | 0.0409 | -1.0900 | down |
| 18 | MEDN2368 | LPE(20:1/0:0) | GP | 2.4052 | 0.0096 | -1.1491 | down |
| 19 | MEDN2370 | LPE(22:2/0:0) | GP | 2.5824 | 0.0050 | -1.5396 | down |
| 20 | MEDN2371 | LPE(22:3/0:0) | GP | 2.6402 | 0.0025 | -1.1171 | down |
| 21 | MEDN2284 | LPE(O-18:2) | GP | 2.3539 | 0.0405 | -0.7330 | down |
| 22 | MEDP1996 | N-Formylglycine | Amino acid and Its metabolites | 2.6062 | 0.0198 | -0.2905 | down |
| 23 | MEDN2225 | PS(18:0/20:4) | GP | 2.3554 | 0.0425 | -0.5551 | down |
| 24 | MEDN2287 | alpha-Methoxy-1H-indole-3-propanoic acid | Heterocyclic compounds | 2.5421 | 0.0182 | 0.2229 | up |

**Supplementary Material Table 4**

**Differential metabolites between the WT and Tas2r105 KO mice**

| NO. | Compounds | RT (min) | Ion | m/z | Formula |
| --- | --- | --- | --- | --- | --- |
| MEDP2265 | 2-Amino-3-phosphonopropionic acid | 0.77 | [M+H]^+^ | 170.0 | C_3_H_8_NO_5_P |
| MEDP2667 | 3-Aminophenol | 1.21 | [M+H]^+^ | 110.1 | C_6_H_7_NO |
| MEDP1132 | 3-Carboxypropyltrimethylammonium | 0.75 | [M+H]^+^ | 146.1 | C_7_H_15_NO_2_ |
| MEDN1509 | 4,6-Diamino-5-formamidopyrimidine | 0.73 | [M-H]^-^ | 152.1 | C_5_H_7_N_5_O |
| MEDP1732 | 4-Methyl-1-phenyl-2-pentanone | 7.84 | [M+H]^+^ | 177.1 | C_12_H_16_O |
| MEDP1202 | 5'-Deoxy-5'-fluoroadenosine | 1.70 | [M+H]^+^ | 270.1 | C_10_H_12_FN_5_O_3_ |
| MEDP0540 | 7,8-dihydro-L-Biopterin | 1.21 | [M+H]^+^ | 240.1 | C_9_H_13_N_5_O_3_ |
| MEDP1002 | Acetylcholine | 0.73 | [M+H]^+^ | 146.1 | C_7_H_15_NO_2_ |
| MEDP0160 | Adenosine | 1.70 | [M+H]^+^ | 268.1 | C_10_H_13_N_5_O_4_ |
| MEDP1499 | All-trans-Retinal | 11.68 | [M+H]^+^ | 285.0 | C_20_H_28_O |
| MEDP0390 | Arainosine | 1.70 | [M+H]^+^ | 269.1 | C_10_H_12_N_4_O_5_ |
| MEDP2326 | Cyclocreatine | 0.44 | [M+H]^+^ | 144.1 | C_5_H_9_N_3_O_2_ |
| MEDN2295 | Estradiol cypionate | 7.22 | [M-H]^-^ | 395.2 | C_26_H_36_O_3_ |
| MEDN0385 | FFA(12:0) | 9.42 | [M-H]^-^ | 199.2 | C_12_H_24_O_2_ |
| MEDN0112 | Glycocholic Acid | 5.91 | [M-H]^-^ | 464.3 | C_26_H_43_NO_6_ |
| MEDP2068 | Hexaethylene-glycol | 2.71 | [M+H]^+^ | 283.2 | C_12_H_26_O_7_ |
| MEDN2367 | LPE(0:0/20:1) | 9.65 | [M-H]^-^ | 506.3 | C_25_H_50_NO_7_P |
| MEDN2368 | LPE(20:1/0:0) | 9.85 | [M-H]^-^ | 506.3 | C_25_H_50_NO_7_P |
| MEDN2370 | LPE(22:2/0:0) | 10.20 | [M-H]^-^ | 532.3 | C_27_H_52_NO_7_P |
| MEDN2371 | LPE(22:3/0:0) | 9.44 | [M-H]^-^ | 530.3 | C_27_H_50_NO_7_P |
| MEDN2284 | LPE(O-18:2) | 9.11 | [M-H]^-^ | 462.3 | C_23_H_46_NO_6_P |
| MEDP1996 | N-Formylglycine | 0.73 | [M+H]^+^ | 104.0 | C_3_H_5_NO_3_ |
| MEDN2225 | PS(18:0/20:4) | 12.30 | [M-H]^-^ | 810.5 | C_44_H_78_NO_10_P |
| MEDN2287 | alpha-Methoxy-1H-indole-3-propanoic acid | 5.63 | [M-H]^-^ | 218.1 | C_12_H_13_NO_3_ |

**Supplementary Material Table 5**

**Correlations between the gut microbiota and metabolites in Tas2r105 KO mice**

| **No.** | **Taxonomy** | **Correlation** | **P-value** | **Compounds** |
| --- | --- | --- | --- | --- |
| 1 | *p__Firmicutes;c__Bacilli;o__Erysipelotrichales;f__unidentified_Erysipelotrichales;g__Erysipelatoclostridium;s__Clostridium_cocleatum* | 0.8117 | 0.0499 | 2-Amino-3-phosphonopropionic acid |
| 2 | *p__Bacteroidota;c__Bacteroidia;o__Bacteroidales;f__Muribaculaceae;g__unidentified_Muribaculaceae;s__mouse_gut_metagenome* | -0.9411 | 0.0051 | 2-Amino-3-phosphonopropionic acid |
| 3 | *p__Bacteroidota;c__Bacteroidia;o__Bacteroidales;f__Muribaculaceae;g__unidentified_Muribaculaceae;s__mouse_gut_metagenome* | 0.8804 | 0.0206 | 3-Aminophenol |
| 4 | *p__Proteobacteria;c__Gammaproteobacteria;o__Pseudomonadales;f__Pseudomonadaceae;g__Pseudomonas;s__bioreactor_metagenome* | -0.8281 | 0.0418 | 3-Aminophenol |
| 5 | *p__unidentified_Bacteria;c__unidentified_Bacteria;o__unidentified_Bacteria;f__unidentified_Bacteria;g__unidentified_Bacteria;s__TM7_phylum_sp_oral_clone_CW040* | -0.8857 | 0.0188 | 3-Carboxypropyltrimethylammonium |
| 6 | *p__Actinobacteria;c__unidentified_Actinobacteria;o__Corynebacteriales;f__Corynebacteriaceae;g__Corynebacterium;s__Corynebacterium_urealyticum* | 0.9276 | 0.0077 | 3-Carboxypropyltrimethylammonium |
| 7 | *p__Firmicutes;c__Clostridia;o__Eubacteriales;f__Eubacteriaceae;g__Eubacterium;s__Eubacterium_limosum* | 0.8697 | 0.0244 | 3-Carboxypropyltrimethylammonium |
| 8 | *p__Bacteroidota;c__Bacteroidia;o__Bacteroidales;f__Muribaculaceae;g__unidentified_Muribaculaceae;s__mouse_gut_metagenome* | -0.9411 | 0.0051 | 4,6-Diamino-5-formamidopyrimidine |
| 9 | *p__Proteobacteria;c__Gammaproteobacteria;o__Pseudomonadales;f__Pseudomonadaceae;g__Pseudomonas;s__bioreactor_metagenome* | 0.8281 | 0.0418 | 4,6-Diamino-5-formamidopyrimidine |
| 10 | *p__Bacteroidota;c__Bacteroidia;o__Bacteroidales;f__Tannerellaceae;g__Parabacteroides;s__Parabacteroides_goldsteinii* | 0.8197 | 0.0458 | 4,6-Diamino-5-formamidopyrimidine |
| 11 | *p__Firmicutes;c__Clostridia;o__unidentified_Clostridia;f__Christensenellaceae;g__unidentified_Christensenellaceae;s__bacterium_YE57* | 0.8452 | 0.0341 | 4,6-Diamino-5-formamidopyrimidine |
| 12 | *p__Bacteroidota;c__Bacteroidia;o__Bacteroidales;f__Bacteroidaceae;g__Bacteroides;s__Bacteroides_acidifaciens* | -0.8286 | 0.0416 | 4-Methyl-1-phenyl-2-pentanone |
| 13 | *p__Bacteroidota;c__Bacteroidia;o__Bacteroidales;f__Muribaculaceae;g__unidentified_Muribaculaceae;s__mouse_gut_metagenome* | -0.8804 | 0.0206 | 4-Methyl-1-phenyl-2-pentanone |
| 14 | *p__Bacteroidota;c__Bacteroidia;o__Flavobacteriales;f__Flavobacteriaceae;g__unidentified_Flavobacteriaceae;s__gut_metagenome* | 0.8452 | 0.0341 | 4-Methyl-1-phenyl-2-pentanone |
| 15 | *p__Firmicutes;c__Bacilli;o__unidentified_Bacilli;f__Paenibacillaceae;g__Xylanibacillus;s__Xylanibacillus_composti* | -0.8281 | 0.0418 | 4-Methyl-1-phenyl-2-pentanone |
| 16 | *p__Firmicutes;c__Clostridia;o__unidentified_Clostridia;f__Oscillospiraceae;g__unidentified_Oscillospiraceae;s__Clostridium_sp_Culture_1* | -0.9429 | 0.0048 | 5'-Deoxy-5'-fluoroadenosine |
| 17 | *p__Bacteroidota;c__Bacteroidia;o__Bacteroidales;f__Muribaculaceae;g__unidentified_Muribaculaceae;s__mouse_gut_metagenome* | -0.8804 | 0.0206 | 5'-Deoxy-5'-fluoroadenosine |
| 18 | *p__Proteobacteria;c__Gammaproteobacteria;o__Pseudomonadales;f__Pseudomonadaceae;g__Pseudomonas;s__bioreactor_metagenome* | 0.8281 | 0.0418 | 5'-Deoxy-5'-fluoroadenosine |
| 19 | *p__Firmicutes;c__Bacilli;o__unidentified_Bacilli;f__Paenibacillaceae;g__Xylanibacillus;s__Xylanibacillus_composti* | -0.8281 | 0.0418 | 5'-Deoxy-5'-fluoroadenosine |
| 20 | *p__unidentified_Bacteria;c__unidentified_Bacteria;o__unidentified_Bacteria;f__unidentified_Bacteria;g__unidentified_Bacteria;s__TM7_phylum_sp_oral_clone_CW040* | -0.9429 | 0.0048 | 7,8-dihydro-L-Biopterin |
| 21 | *p__Firmicutes;c__Clostridia;o__unidentified_Clostridia;f__Oscillospiraceae;g__unidentified_Oscillospiraceae;s__Clostridium_sp_Culture_1* | -0.8286 | 0.0416 | 7,8-dihydro-L-Biopterin |
| 22 | *p__Proteobacteria;c__Gammaproteobacteria;o__Pseudomonadales;f__Pseudomonadaceae;g__Pseudomonas;s__bioreactor_metagenome* | 0.8281 | 0.0418 | 7,8-dihydro-L-Biopterin |
| 23 | *p__Firmicutes;c__Bacilli;o__unidentified_Bacilli;f__Paenibacillaceae;g__Xylanibacillus;s__Xylanibacillus_composti* | -0.8281 | 0.0418 | 7,8-dihydro-L-Biopterin |
| 24 | *p__Firmicutes;c__Clostridia;o__unidentified_Clostridia;f__Christensenellaceae;g__unidentified_Christensenellaceae;s__bacterium_YE57* | 0.8452 | 0.0341 | 7,8-dihydro-L-Biopterin |
| 25 | *p__unidentified_Bacteria;c__unidentified_Bacteria;o__unidentified_Bacteria;f__unidentified_Bacteria;g__unidentified_Bacteria;s__TM7_phylum_sp_oral_clone_CW040* | -0.8286 | 0.0416 | Acetylcholine |
| 26 | *p__Firmicutes;c__Clostridia;o__unidentified_Clostridia;f__Oscillospiraceae;g__unidentified_Oscillospiraceae;s__Clostridium_sp_Culture_1* | -0.8857 | 0.0188 | Acetylcholine |
| 27 | *p__Bacteroidota;c__Bacteroidia;o__Bacteroidales;f__Muribaculaceae;g__unidentified_Muribaculaceae;s__mouse_gut_metagenome* | -0.8804 | 0.0206 | Acetylcholine |
| 28 | *p__Proteobacteria;c__Gammaproteobacteria;o__Pseudomonadales;f__Pseudomonadaceae;g__Pseudomonas;s__bioreactor_metagenome* | 0.8281 | 0.0418 | Acetylcholine |
| 29 | *p__Firmicutes;c__Bacilli;o__unidentified_Bacilli;f__Paenibacillaceae;g__Xylanibacillus;s__Xylanibacillus_composti* | -0.8281 | 0.0418 | Acetylcholine |
| 30 | *p__Firmicutes;c__Clostridia;o__unidentified_Clostridia;f__Christensenellaceae;g__unidentified_Christensenellaceae;s__bacterium_YE57* | 0.8452 | 0.0341 | Acetylcholine |
| 31 | *p__Firmicutes;c__Clostridia;o__unidentified_Clostridia;f__Oscillospiraceae;g__unidentified_Oscillospiraceae;s__Clostridium_sp_Culture_1* | -0.8286 | 0.0416 | Adenosine |
| 32 | *p__Bacteroidota;c__Bacteroidia;o__Bacteroidales;f__Muribaculaceae;g__unidentified_Muribaculaceae;s__mouse_gut_metagenome* | -0.9411 | 0.0051 | Adenosine |
| 33 | *p__Proteobacteria;c__Gammaproteobacteria;o__Pseudomonadales;f__Pseudomonadaceae;g__Pseudomonas;s__bioreactor_metagenome* | 0.8281 | 0.0418 | Adenosine |
| 34 | *p__Firmicutes;c__Clostridia;o__unidentified_Clostridia;f__Oscillospiraceae;g__unidentified_Oscillospiraceae;s__Clostridium_sp_Culture_1* | 0.8286 | 0.0416 | All-trans-Retinal |
| 35 | *p__Bacteroidota;c__Bacteroidia;o__Bacteroidales;f__Muribaculaceae;g__unidentified_Muribaculaceae;s__mouse_gut_metagenome* | 0.8804 | 0.0206 | All-trans-Retinal |
| 36 | *p__Firmicutes;c__Bacilli;o__unidentified_Bacilli;f__Paenibacillaceae;g__Xylanibacillus;s__Xylanibacillus_composti* | 0.8281 | 0.0418 | All-trans-Retinal |
| 37 | *p__Firmicutes;c__Bacilli;o__Erysipelotrichales;f__unidentified_Erysipelotrichales;g__Erysipelatoclostridium;s__Clostridium_cocleatum* | 0.8986 | 0.0149 | Arainosine |
| 38 | *p__Firmicutes;c__Negativicutes;o__Acidaminococcales;f__Acidaminococcaceae;g__Phascolarctobacterium;s__Phascolarctobacterium_faecium* | -0.8197 | 0.0458 | Arainosine |
| 39 | *p__Proteobacteria;c__Alphaproteobacteria;o__Rhizobiales;f__Beijerinckiaceae;g__Chelatococcus;s__Chelatococcus_daeguensis* | -0.8197 | 0.0458 | Arainosine |
| 40 | *p__Proteobacteria;c__Alphaproteobacteria;o__Rhizobiales;f__Rhizobiaceae;g__Mesorhizobium;s__metagenome* | -0.8197 | 0.0458 | Arainosine |
| 41 | *p__Bacteroidota;c__Bacteroidia;o__Bacteroidales;f__Muribaculaceae;g__unidentified_Muribaculaceae;s__mouse_gut_metagenome* | -0.8804 | 0.0206 | Arainosine |
| 42 | *p__Firmicutes;c__Bacilli;o__unidentified_Bacilli;f__Paenibacillaceae;g__Xylanibacillus;s__Xylanibacillus_composti* | -0.8281 | 0.0418 | Arainosine |
| 43 | *p__Bacteroidota;c__Bacteroidia;o__Bacteroidales;f__Muribaculaceae;g__unidentified_Muribaculaceae;s__mouse_gut_metagenome* | -0.9411 | 0.0051 | Cyclocreatine |
| 44 | *p__Proteobacteria;c__Gammaproteobacteria;o__Enterobacterales;f__Morganellaceae;g__Proteus;s__Proteus_mirabilis* | 0.8452 | 0.0341 | Cyclocreatine |
| 45 | *p__Bacteroidota;c__Bacteroidia;o__Bacteroidales;f__Bacteroidaceae;g__Bacteroides;s__Bacteroides_ovatus* | 0.8452 | 0.0341 | Cyclocreatine |
| 46 | *p__unidentified_Bacteria;c__unidentified_Bacteria;o__unidentified_Bacteria;f__unidentified_Bacteria;g__unidentified_Bacteria;s__TM7_phylum_sp_oral_clone_CW040* | 0.8286 | 0.0416 | Estradiol cypionate |
| 47 | *p__Bacteroidota;c__Bacteroidia;o__Bacteroidales;f__Bacteroidaceae;g__Bacteroides;s__Bacteroides_acidifaciens* | 0.9429 | 0.0048 | Estradiol cypionate |
| 48 | *p__unidentified_Bacteria;c__unidentified_Bacteria;o__unidentified_Bacteria;f__unidentified_Bacteria;g__unidentified_Bacteria;s__TM7_phylum_sp_oral_clone_CW040* | -0.9429 | 0.0048 | FFA(12:0) |
| 49 | *p__Bacteroidota;c__Bacteroidia;o__Bacteroidales;f__Bacteroidaceae;g__Bacteroides;s__Bacteroides_acidifaciens* | -0.8286 | 0.0416 | FFA(12:0) |
| 50 | *p__Proteobacteria;c__Gammaproteobacteria;o__Pseudomonadales;f__Pseudomonadaceae;g__Pseudomonas;s__bioreactor_metagenome* | 0.8281 | 0.0418 | FFA(12:0) |
| 51 | *p__Bacteroidota;c__Bacteroidia;o__Bacteroidales;f__Muribaculaceae;g__unidentified_Muribaculaceae;s__mouse_gut_metagenome* | 0.8804 | 0.0206 | Glycocholic Acid |
| 52 | *p__Proteobacteria;c__Gammaproteobacteria;o__Enterobacterales;f__Morganellaceae;g__Proteus;s__Proteus_mirabilis* | -0.8452 | 0.0341 | Glycocholic Acid |
| 53 | *p__Bacteroidota;c__Bacteroidia;o__Bacteroidales;f__Bacteroidaceae;g__Bacteroides;s__Bacteroides_ovatus* | -0.8452 | 0.0341 | Glycocholic Acid |
| 54 | *p__Bacteroidota;c__Bacteroidia;o__Bacteroidales;f__Muribaculaceae;g__unidentified_Muribaculaceae;s__mouse_gut_metagenome* | 0.9411 | 0.0051 | Hexaethylene-glycol |
| 55 | *p__Proteobacteria;c__Gammaproteobacteria;o__Pseudomonadales;f__Pseudomonadaceae;g__Pseudomonas;s__bioreactor_metagenome* | -0.8281 | 0.0418 | Hexaethylene-glycol |
| 56 | *p__Bacteroidota;c__Bacteroidia;o__Bacteroidales;f__Tannerellaceae;g__Parabacteroides;s__Parabacteroides_goldsteinii* | -0.8197 | 0.0458 | Hexaethylene-glycol |
| 57 | *p__Firmicutes;c__Clostridia;o__unidentified_Clostridia;f__Christensenellaceae;g__unidentified_Christensenellaceae;s__bacterium_YE57* | -0.8452 | 0.0341 | Hexaethylene-glycol |
| 58 | *p__Bacteroidota;c__Bacteroidia;o__Bacteroidales;f__Bacteroidaceae;g__Bacteroides;s__Bacteroides_acidifaciens* | -0.9429 | 0.0048 | LPE(0:0/20:1) |
| 59 | *p__Bacteroidota;c__Bacteroidia;o__Flavobacteriales;f__Flavobacteriaceae;g__unidentified_Flavobacteriaceae;s__gut_metagenome* | 0.8452 | 0.0341 | LPE(0:0/20:1) |
| 60 | *p__Firmicutes;c__Bacilli;o__unidentified_Bacilli;f__Paenibacillaceae;g__Xylanibacillus;s__Xylanibacillus_composti* | -0.8281 | 0.0418 | LPE(0:0/20:1) |
| 61 | *p__Bacteroidota;c__Bacteroidia;o__Bacteroidales;f__Bacteroidaceae;g__Bacteroides;s__Bacteroides_acidifaciens* | -0.9429 | 0.0048 | LPE(20:1/0:0) |
| 62 | *p__Bacteroidota;c__Bacteroidia;o__Flavobacteriales;f__Flavobacteriaceae;g__unidentified_Flavobacteriaceae;s__gut_metagenome* | 0.8452 | 0.0341 | LPE(20:1/0:0) |
| 63 | *p__Firmicutes;c__Bacilli;o__unidentified_Bacilli;f__Paenibacillaceae;g__Xylanibacillus;s__Xylanibacillus_composti* | -0.8281 | 0.0418 | LPE(20:1/0:0) |
| 64 | *p__unidentified_Bacteria;c__unidentified_Bacteria;o__unidentified_Bacteria;f__unidentified_Bacteria;g__unidentified_Bacteria;s__TM7_phylum_sp_oral_clone_CW040* | -0.8286 | 0.0416 | LPE(22:2/0:0) |
| 65 | *p__Bacteroidota;c__Bacteroidia;o__Bacteroidales;f__Bacteroidaceae;g__Bacteroides;s__Bacteroides_acidifaciens* | -0.9429 | 0.0048 | LPE(22:2/0:0) |
| 66 | *p__Actinobacteria;c__unidentified_Actinobacteria;o__Corynebacteriales;f__Corynebacteriaceae;g__Corynebacterium;s__Corynebacterium_urealyticum* | 0.8697 | 0.0244 | LPE(22:2/0:0) |
| 67 | *p__Firmicutes;c__Clostridia;o__Eubacteriales;f__Eubacteriaceae;g__Eubacterium;s__Eubacterium_limosum* | 0.9276 | 0.0077 | LPE(22:2/0:0) |
| 68 | *p__Proteobacteria;c__Gammaproteobacteria;o__Enterobacterales;f__Morganellaceae;g__Proteus;s__Proteus_mirabilis* | 0.8452 | 0.0341 | LPE(22:2/0:0) |
| 69 | *p__Bacteroidota;c__Bacteroidia;o__Bacteroidales;f__Bacteroidaceae;g__Bacteroides;s__Bacteroides_ovatus* | 0.8452 | 0.0341 | LPE(22:2/0:0) |
| 70 | *p__unidentified_Bacteria;c__unidentified_Bacteria;o__unidentified_Bacteria;f__unidentified_Bacteria;g__unidentified_Bacteria;s__TM7_phylum_sp_oral_clone_CW040* | -0.9429 | 0.0048 | LPE(22:3/0:0) |
| 71 | *p__Firmicutes;c__Clostridia;o__unidentified_Clostridia;f__Oscillospiraceae;g__unidentified_Oscillospiraceae;s__Clostridium_sp_Culture_1* | -0.8286 | 0.0416 | LPE(22:3/0:0) |
| 72 | *p__Proteobacteria;c__Gammaproteobacteria;o__Pseudomonadales;f__Pseudomonadaceae;g__Pseudomonas;s__bioreactor_metagenome* | 0.8281 | 0.0418 | LPE(22:3/0:0) |
| 73 | *p__Firmicutes;c__Bacilli;o__unidentified_Bacilli;f__Paenibacillaceae;g__Xylanibacillus;s__Xylanibacillus_composti* | -0.8281 | 0.0418 | LPE(22:3/0:0) |
| 74 | *p__Firmicutes;c__Clostridia;o__unidentified_Clostridia;f__Christensenellaceae;g__unidentified_Christensenellaceae;s__bacterium_YE57* | 0.8452 | 0.0341 | LPE(22:3/0:0) |
| 75 | *p__Bacteroidota;c__Bacteroidia;o__Bacteroidales;f__Bacteroidaceae;g__Bacteroides;s__Bacteroides_acidifaciens* | -0.8857 | 0.0188 | LPE(O-18:2) |
| 76 | *p__Actinobacteria;c__unidentified_Actinobacteria;o__Corynebacteriales;f__Corynebacteriaceae;g__Corynebacterium;s__Corynebacterium_urealyticum* | 0.8697 | 0.0244 | LPE(O-18:2) |
| 77 | *p__Firmicutes;c__Clostridia;o__Eubacteriales;f__Eubacteriaceae;g__Eubacterium;s__Eubacterium_limosum* | 0.9276 | 0.0077 | LPE(O-18:2) |
| 78 | *p__Proteobacteria;c__Gammaproteobacteria;o__Enterobacterales;f__Morganellaceae;g__Proteus;s__Proteus_mirabilis* | 0.8452 | 0.0341 | LPE(O-18:2) |
| 79 | *p__Bacteroidota;c__Bacteroidia;o__Bacteroidales;f__Bacteroidaceae;g__Bacteroides;s__Bacteroides_ovatus* | 0.8452 | 0.0341 | LPE(O-18:2) |
| 80 | *p__unidentified_Bacteria;c__unidentified_Bacteria;o__unidentified_Bacteria;f__unidentified_Bacteria;g__unidentified_Bacteria;s__TM7_phylum_sp_oral_clone_CW040* | -1.0000 | 0.0000 | N-Formylglycine |
| 81 | *p__Actinobacteria;c__unidentified_Actinobacteria;o__Corynebacteriales;f__Corynebacteriaceae;g__Corynebacterium;s__Corynebacterium_urealyticum* | 0.8117 | 0.0499 | N-Formylglycine |
| 82 | *p__Proteobacteria;c__Gammaproteobacteria;o__Pseudomonadales;f__Pseudomonadaceae;g__Pseudomonas;s__bioreactor_metagenome* | 0.8281 | 0.0418 | N-Formylglycine |
| 83 | *p__Firmicutes;c__Clostridia;o__unidentified_Clostridia;f__Christensenellaceae;g__unidentified_Christensenellaceae;s__bacterium_YE57* | 0.8452 | 0.0341 | N-Formylglycine |
| 84 | *p__Bacteroidota;c__Bacteroidia;o__Bacteroidales;f__Muribaculaceae;g__unidentified_Muribaculaceae;s__mouse_gut_metagenome* | -0.9411 | 0.0051 | PS(18:0/20:4) |
| 85 | *p__Proteobacteria;c__Gammaproteobacteria;o__Enterobacterales;f__Morganellaceae;g__Proteus;s__Proteus_mirabilis* | 0.8452 | 0.0341 | PS(18:0/20:4) |
| 86 | *p__Bacteroidota;c__Bacteroidia;o__Bacteroidales;f__Bacteroidaceae;g__Bacteroides;s__Bacteroides_ovatus* | 0.8452 | 0.0341 | PS(18:0/20:4) |
| 87 | *p__Firmicutes;c__Bacilli;o__Erysipelotrichales;f__unidentified_Erysipelotrichales;g__Erysipelatoclostridium;s__Clostridium_cocleatum* | -0.8407 | 0.0361 | alpha-Methoxy-1H-indole-3-propanoic acid |
| 88 | *p__Firmicutes;c__Negativicutes;o__Acidaminococcales;f__Acidaminococcaceae;g__Phascolarctobacterium;s__Phascolarctobacterium_faecium* | 0.8197 | 0.0458 | alpha-Methoxy-1H-indole-3-propanoic acid |
| 89 | *p__Proteobacteria;c__Alphaproteobacteria;o__Rhizobiales;f__Beijerinckiaceae;g__Chelatococcus;s__Chelatococcus_daeguensis* | 0.8197 | 0.0458 | alpha-Methoxy-1H-indole-3-propanoic acid |
| 90 | *p__Proteobacteria;c__Alphaproteobacteria;o__Rhizobiales;f__Rhizobiaceae;g__Mesorhizobium;s__metagenome* | 0.8197 | 0.0458 | alpha-Methoxy-1H-indole-3-propanoic acid |
| 91 | *p__Bacteroidota;c__Bacteroidia;o__Bacteroidales;f__Muribaculaceae;g__unidentified_Muribaculaceae;s__mouse_gut_metagenome* | 0.8804 | 0.0206 | alpha-Methoxy-1H-indole-3-propanoic acid |
| 92 | *p__Proteobacteria;c__Gammaproteobacteria;o__Enterobacterales;f__Morganellaceae;g__Proteus;s__Proteus_mirabilis* | -0.8452 | 0.0341 | alpha-Methoxy-1H-indole-3-propanoic acid |
| 93 | *p__Firmicutes;c__Bacilli;o__unidentified_Bacilli;f__Paenibacillaceae;g__Xylanibacillus;s__Xylanibacillus_composti* | 0.8281 | 0.0418 | alpha-Methoxy-1H-indole-3-propanoic acid |
| 94 | *p__Bacteroidota;c__Bacteroidia;o__Bacteroidales;f__Bacteroidaceae;g__Bacteroides;s__Bacteroides_ovatus* | -0.8452 | 0.0341 | alpha-Methoxy-1H-indole-3-propanoic acid |

**Supplementary Material Figure 1**

Individual extracted MRM scans for the 24 differential metabolites


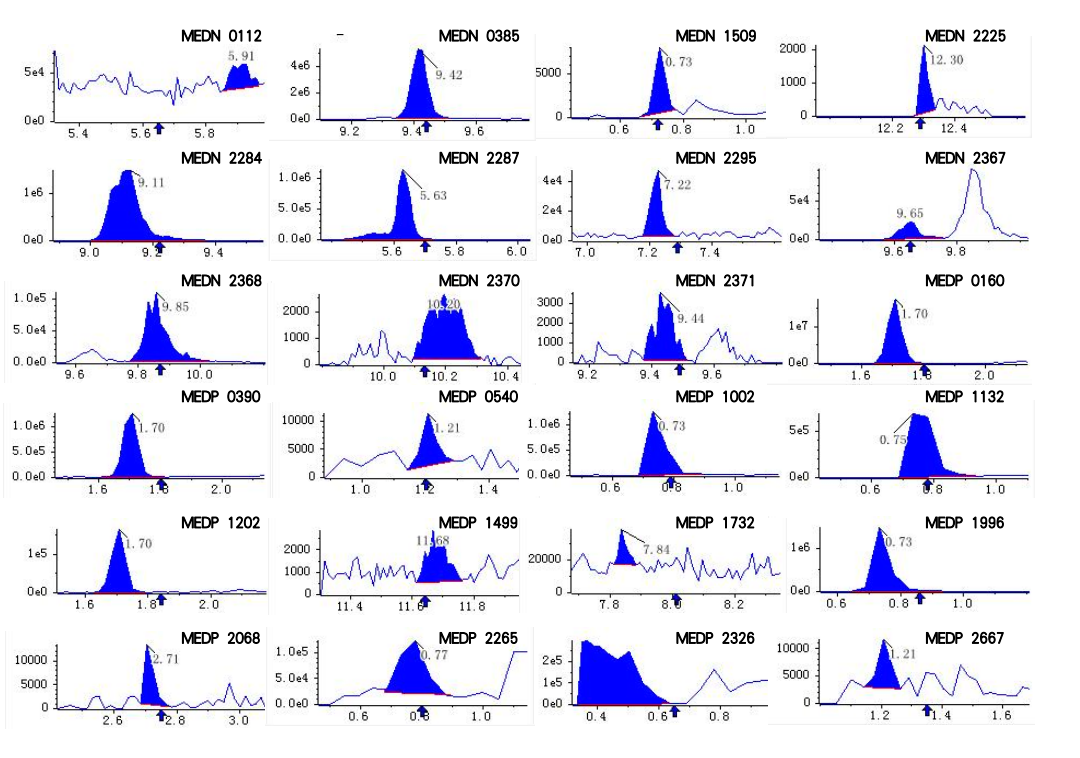

Supplement: Supplemental material — Supplemental tables and figure. [file msystems.01556-24-s0001.docx]
